# Aetiological factors in functional seizures and functional motor symptoms: shared and distinct features

**DOI:** 10.1136/jnnp-2025-337694

**Published:** 2026-05-07

**Authors:** L S Merritt Millman, Snigdha Bhuma, Yasmine Basamh, Jemima Uloyok-Job, Jessica Davies, Lauren Blunstone, Akshaana Manoharan, Jan A Coebergh, Anthony S David, Mark J Edwards, Laura H Goldstein, John Hodsoll, Mitul A Mehta, Timothy R Nicholson, Biba Stanton, Joel S Winston, Trudie Chalder, Matthew Hotopf, Susannah Pick

**Affiliations:** 1 King's College London, Institute of Psychiatry, Psychology and Neuroscience, London, UK; 2 South London and Maudsley NHS Foundation Trust, London, UK; 3 Neurology, St George’s University Hospitals NHS Foundation Trust, London, England, UK; 4 Institute of Mental Health, Division of Psychiatry, University College London, London, UK; 5 King’s College Hospital NHS Foundation Trust, London, UK

**Keywords:** FUNCTIONAL NEUROLOGICAL DISORDER, NEUROPSYCHIATRY, PSYCHIATRY, QUALITY OF LIFE

## Abstract

**Background:**

Functional seizures (FS) and functional motor symptoms (FMS), subtypes of functional neurological disorder, may involve shared and distinct predisposing, precipitating, perpetuating and triggering (PPPT) factors. This study investigated potential self-reported PPPT factors in FS and FMS separately.

**Methods:**

200 participants (FS=50, FMS=50, individuals with anxiety and/or depression (clinical controls [CC]=50, healthy controls [HC]=50) completed an in-depth medical history interview and online questionnaires to assess potential aetiological factors including traumatic/adverse life events, alexithymia, autistic traits, psychological and physical symptoms, illness perceptions, cognitive-behavioural responses and outcome measures of general functioning and health-related quality of life (HRQoL).

**Results:**

Participants with FS more frequently reported traumatic/adverse events and psychopathology (eg, dissociation, posttraumatic stress disorder) as possible illness causes/precipitating factors and sensory symptom triggers, relative to FMS and/or CCs. In contrast, participants with FMS endorsed physical illness causes/precipitating factors and physical activity and emotion-related symptom triggers more frequently than FS and/or CCs. Physical and dissociative symptoms were elevated, alongside reductions in HRQoL and general functioning in FS/FMS compared with CCs/HCs. Greater current, threatening illness-related beliefs/cognitions were disclosed in FS/FMS compared with CCs. Negative associations between HRQoL, cognitive-behavioural responses and traumatic/adverse events were also seen in FS/FMS.

**Conclusions:**

Traumatic events and psychopathology may be more prominently involved in the development/maintenance of FS and physical illness/injury, physical functioning and alexithymia may be more central to FMS. Unhelpful illness-related beliefs and impacts on HRQoL and general functioning are shared between these subtypes. The results of this study provide insight into potential unique and overlapping characteristics of FS/FMS, with implications for treatment.

**WHAT IS ALREADY KNOWN ON THIS TOPIC**

- Functional seizures (FS) and functional motor symptoms (FMS), two common subtypes of functional neurological disorder, may involve both intersecting and independent predisposing, precipitating, perpetuating and triggering (PPPT) factors. To date, no original large-scale research has extensively directly compared PPPT factors in FS and FMS.

**WHAT THIS STUDY ADDS**

- This study provides evidence for possible subtype differences in self-reported characteristics in FS and FMS: past trauma, psychopathology and sensory symptom triggers were more commonly reported in FS, while physical illness and physical and emotion-related symptom triggers were more often reported in FMS. Impairments in general functioning and health-related quality of life were similar in FS and FMS, and threatening illness-related beliefs and perceptions, which may play a significant perpetuating role, were also expressed in both groups.

**HOW THIS STUDY MIGHT AFFECT RESEARCH, PRACTICE OR POLICY**

- Our findings regarding shared and distinct PPPT factors in FS and FMS may facilitate the development of individualised or subtype-specific treatment approaches and could guide the optimisation of therapeutic environments.

## Introduction

 Psychological, social and biological factors are known to contribute to the aetiology of functional neurological disorder (FND),^1^ including adverse life events/trauma, psychological disorders, physical injury/accidents and social/environmental challenges.^2 3^ The disorder often results in impairments in health-related quality of life (HRQoL) and the experience of diverse psychological and physical symptoms.^4^

FND involves a range of symptom presentations (eg, weakness, tremor, seizures, sensory issues), which may be mixed and/or begin as one symptom type and develop into another over time.^5 6^ Within FND, there are also individual subtypes, two of the most common being functional seizures (FS, characterised by events that resemble epileptic seizures—without electroencephalogram correlates—or syncope) and functional motor symptoms (FMS, characterised by disturbances in voluntary motor functions).^2^Previous research has indicated both potential similarities^7–9^ and differences^10 11^ in the clinical characteristics and aetiological factors that may play a role in the development or maintenance of FS and FMS. Although both subtypes often include elevations in pain or the diagnosis of other somatic disorders (eg, fibromyalgia) and mental health difficulties (eg, depression and anxiety),^7 8^ elevated dissociative symptoms and adverse life events, including childhood abuse, have been more specifically reported in FS.^9–11^ Given the current literature and possibility that these subtypes may involve different aetiological factors,^10^ further research is required to understand patients’ perspectives on the independent and/or overlapping factors that may predispose, precipitate, maintain or trigger symptoms in these subtypes (box 1). This enhanced understanding will have implications for treatment and outcomes.^6^

Box 1 **Definitions of predisposing, precipitating, perpetuating and triggering factors**

*Predisposing:* factors that may increase vulnerability to developing symptoms (eg, psychosocial adversity including abuse/trauma, physical or psychological factors).

*Precipitating:* self-reported factors occurring within 12 months before symptoms started that may have initiated disorder onset (eg, traumatic/adverse events, physical injury, mental illness).

*Perpetuating: *factors that may maintain the presence of symptoms (eg, illness beliefs, cognitive behavioural responses).

*Triggering:* perceived factors that make symptoms occur or get worse in the short term, on an ongoing basis (eg, bright lights, pain, physical exertion, reminders of traumatic events).

As part of a larger research programme investigating psychobiological causes and mechanisms in FS and FMS (https://osf.io/y834q),^12^ this study investigated possible self-reported aetiological factors and symptom triggers in these subgroups, compared with clinical control (individuals with a diagnosis of anxiety and/or depression; CC) and healthy control (HC) groups.

The following hypotheses were formulated^12^:

1. Elevated depressive symptoms and anxiety in FS/FMS relative to HCs and more other physical symptoms, alexithymia, autistic traits, somatoform and psychological dissociation in FS and/or FMS compared with HCs/CCs.^3 4 13–15^
2. A greater number and heightened impact of adverse events and elevated post-traumatic symptoms and diagnoses would be reported in FS compared to all other groups and in FMS compared with HCs/CCs.^3 16 17^
3. Reduced work and social functioning and worse HRQoL would be reported in FS/FMS compared to HCs/CCs.^4^
4. Perceived causes of illness in FS and FMS would include physical factors (illness/injury), stress/emotion, work-related factors and adverse life events.
5. Perceived symptom triggers in the FND groups would include physical activity/exertion, fatigue, emotions/stress and sensory factors.^4 18^
6. Both FND groups would report more maladaptive illness-related beliefs and behaviours than CCs.^4^

## Methods

### Study design and participants

This case-control study was sponsored by King’s College London (KCL), conducted in compliance with the World Medical Association Declaration of Helsinki (1996) and was approved by the North-West Greater Manchester South Research Ethics Committee (ref NW/23/0217). Recruitment began in November 2023, with data collected between December 2023 and March 2025, and all study activities organised from the Institute of Psychiatry, Psychology and Neuroscience, KCL.

Participants with FS and FMS were referred from neurology and/or neuropsychiatry services across King’s College Hospital, St George’s University Hospital and South London and Maudsley NHS Foundation Trusts. Participants in the FND groups were also recruited via advertisements online by FND Hope UK. CCs and HCs were recruited from the community using advertisements in public forums. CC participants were also recruited via some existing research cohorts at KCL. Full details of the consent and screening procedures are described elsewhere.^12^

A total sample of 180 participants was expected to provide 80% power to detect a small effect (f=0·1) with an alpha of 0·05 for between-group (FS/FMS/CC/HC) Analysis of Variance (ANOVAs) testing the primary hypotheses.^19^ To allow for non-completion and missing data, 200 participants were recruited.

Eligibility criteria required all participants to be 18–65 years old, with normal or corrected eyesight and fluency in English. Controls across both groups were frequency-matched to FND groups with a 1:1 ratio for sex/gender, age and handedness. Participants in the FND groups were required to meet current Diagnostic and Statistical Manual of Mental Disorders-5 (DSM-5) criteria, with either FS in the absence of FMS or FMS in the absence of FS. Individuals experiencing both symptom types within the previous 12 months were ineligible. In cases where participants volunteered via advertisements, medical documentation was checked to confirm diagnosis (SP). This documentation was required to include a confirmed, stated diagnosis of FND, made by a consultant neurologist/epileptologist (FS) or consultant neurologist (FMS). Where needed, experienced FND clinicians within the research group were consulted for verification.

CCs were required to have a diagnosis of major depression and/or an anxiety disorder, confirmed with DSM-5^1^ criteria during the clinical screening interview (Quick Structured Interview for DSM-5 Disorders).^12^ Exclusion criteria across all groups were having a diagnosis of a significant comorbid neurological (eg, epilepsy) or cardiovascular (eg, heart failure) disorder, the presence of an active, severe psychological illness (eg, alcohol or substance dependence, psychosis), disability or physical symptoms interfering with study task completion (eg, bilateral upper limb paralysis) or current participation in another interventional study (eg, experimental research, treatment trial).

After written informed consent was obtained, participants completed an indepth medical history interview (online supplement) with SP or a trained member of the research team (LSMM, SB).^12^ Eligible participants were emailed a weblink to a set of questionnaires online via Qualtrics (www.qualtrics.com) to complete ahead of attendance at a laboratory session (results presented elsewhere). After completion of the questionnaires and laboratory session, participants were reimbursed with a £50 shopping voucher.

### Measures

Table 1 describes the self-report questionnaires administered at baseline (references included in the online supplemental materials).

**Table 1:** Self-report questionnaires

Questionnaire	Content	Scoring
Functional Neurological Symptoms Questionnaire (see online supplement)^12^	13 items; bespoke questionnaire assessing the presence, frequency, impact, and severity of FND symptoms	Participants report the presence (yes or no), severity (from 1=symptom not present to 7=very severe), frequency (less than weekly to constant) and impact (from 1=no impact at all to 7=very severe impact) of FND symptoms within the past week, with elevated scores indicating more severe, frequent and impactful symptoms; identify the symptom having the most impact on them and the earliest or most consistent premonitory symptom (for individuals with functional seizures only).
Generalised Anxiety Disorder-7^S1^	Seven items; measures generalised anxiety	Symptoms over the past 2 weeks are rated from 0 (‘not at all’) to 3 (‘nearly every day’), with total scores ranging from 0 to 21 (α=0.93) and higher scores indicating elevated anxiety.
Patient Health Questionnaire–9^S2^	Nine items; measures depressive symptoms	Symptoms over the past 2 weeks are rated from 0 (‘not at all’) to 3 (‘nearly every day’), with total scores ranging from 0 to 27 (α=0.92) and higher scores indicating elevated depressive symptomatology.
Patient Health Questionnaire–15^**S3**^	15 items; measures common physical symptoms	How much the participant has been bothered by physical symptoms over the past 4 weeks are rated on a scale from 0 (‘not bothered at all’) to 2 (‘bothered a lot’), with total scores ranging from 0 to 30 (α=0.82).
Multiscale Dissociation Inventory^S4^	30 items; measures psychological dissociative symptoms	Items are rated from 1 (‘never’) to 5 (‘very often’), with subscale scores ranging from 5 to 21 and higher scores indicating elevated dissociative symptoms. Raw scores are converted to T scores prior to analysis. The six subscales are disengagement (five items; α=0.84), depersonalisation (five items; α=0.89), derealisation (five items; α=0.91), memory disturbance (five items; α=0.86), emotional constriction (five items; α=0.95) and identity dissociation (five items; α=0.96).
Somatoform Dissociation Questionnaire–20^**S5**^	20 items; measures bodily dissociative symptoms	Items are rated from 1 (‘this applies to me not at all’) to 5 (‘this applies to me extremely’), with total scores ranging from 20 to 100 (α=0.86) and higher scores indicate more bodily dissociative symptoms. Participants are also asked to indicate if they know the physical cause of each symptom/experience they have endorsed.
Autism-Spectrum Quotient^**S6**^	50 items; measures autistic spectrum traits	For each item, participants rate how strongly they agree (definitely or slightly agree; 1 point) or disagree (slightly or definitely disagree; 0 points). Total (0–50 (α=0.90)) and subscale scores are examined in this study: attention switching (10 items; α=0.72), attention to detail (10 items; α=0.63), social skills (10 items; α=0.79), communication (10 items; α=0.80) and imagination (10 items; α=0.63), with higher scores indicating more autistic spectrum traits.
Toronto Alexithymia Scale-20^**S7**^	20 items; measures difficulty describing and identifying emotions	Items are rated from 1 (‘strongly disagree’) to 5 (‘strongly agree’), with total scores ranging from 20 to 100 (α=0.88) and three subscales measuring difficulty describing feelings (five items; α=0.84), difficulty identifying feelings (seven items; α=0.86) and externally oriented thinking (eight items; α=0.67). Higher scores indicate more alexithymic symptoms.
Traumatic Experiences Checklist^**S8**^	29 items; measures the impact and presence of adverse life events	For each item, participants are asked to indicate whether they have experienced the event (yes or no). If yes, they are then asked at what age/s the event occurred and the level of impact of the event from 1 (‘none’) to 5 (‘an extreme amount’). Total scores (from 0 to 29; α=0.85) as well as impact scores (from 0 to 145) are calculated. Higher scores indicate more traumatic/adverse events and/or a greater impact of events.
Posttraumatic Stress Diagnostic Scale-5^**S9**^	24 items; measures posttraumatic stress disorder symptom severity	Participants are first asked to indicate if they have ever experienced, witnessed or been repeatedly confronted with a list of possible traumas. If yes, participants are then asked to indicate which traumatic experience is currently bothering them the most and respond to 20 statements regarding how often the trauma has been present/how much it has been bothering them over the last month from 0 (‘not at all’) to 4 (‘six or more times a week’ or ‘severe’). Total scores range from 0 to 80 (α=0.96), with a score of 28 used as a cut-off for possible post-traumatic stress disorder (PTSD) diagnosis, and higher scores suggesting more PTSD-related symptoms.
Brief Illness Perception Questionnaire^S10^	Nine items; measures illness beliefs and representations	Consequences, timeline, personal control, treatment control, identity, coherence, emotional representation and concern are each scored from 0 (eg, no effect, no symptoms, no concern) to 10 (eg, severe effect, many severe symptoms, extremely concerned). The ninth item asks participants to rank order the three factors they believe caused their illness.
Cognitive Behavioural Responses Questionnaire^S11^	40 items; measures cognitive and behavioural responses to symptoms	Items are scored on a five-point Likert scale (from 0=strongly agree/never to 4=strongly agree/all the time). Total scores range from 0 to 160 and are examined alongside seven subscales: fear avoidance (four items; α=0.84), catastrophising (four items; α=0.70), damaging beliefs (three items; α=0.78), embarrassment avoidance (three items; α=0.85), symptom focusing (four items; α=0.85), all-or-nothing behaviour (five items; α=0.64) and avoidance/resting behaviour (four items; α=0.69). Elevated total and subscale scores suggest more unhelpful cognitive and behavioural responses to symptoms.
36-Item Short-Form Health Survey^S12^	36 items; measures health-related quality of life	Participants are asked questions regarding their health including limits on one’s ability to complete certain activities, problems resulting from physical or emotional health, pain levels and interference with social activities within the past month. Higher scores (from 0 to 100) suggest better health-related quality of life across eight subscales: physical functioning (10 items; α=0.97), role limitations due to physical health (four items; α=0.93) or emotional problems (three items; α=0.88), energy and fatigue (four items; α=0.88), emotional well-being (five items; α=0.88), social functioning (two items; α=0.90), pain (two items; α=0.90) and general health (five items; α=0.89).
Work and Social Adjustment Scale^S13^	Five items; measures the presence of impairments in social and occupational functioning	Items are rated from 0 (‘not at all’) to 8 (‘severely’), with total scores ranging from 0 to 40 and higher scores indicating elevated impairment (α=0.94).

(References S1–S13 listed in online supplemental materials)

FND, functional neurological disorder.

### Statistical analysis

Data were independently analysed by LSMM and SP using R (V.4.5.1) and SPSS (V.29). Missing data were examined for all outcome variables (online supplemental table S1). In the CC group, two participants did not complete any of the self-report measures, resulting in a total sample size for analysis of 198 participants. Outliers were identified and winsorised (online supplemental table S2), with analyses run with both raw and winsorised values (raw data analyses presented in text, winsorised in online supplemental table S17).

Normality (Shapiro-Wilk test, Q-Q plots) and homogeneity of variance (Bartlett’s test) were evaluated. Categorical variables were analysed with Pearson’s χ^2^ or Fisher’s exact tests, with Cramer’s V as the measure of effect size. Continuous variables were analysed with one-way ANOVA or Welch’s ANOVA (unequal variances) with group (FS/FMS/CC/HC) as the independent factor and eta squared as the measure of effect size. Significant results were followed up with post-hoc tests (pairwise with Holm correction, Games-Howell). Analyses of questionnaires with multiple subscales were run with separate univariate tests and Bonferroni corrected alpha values (Autism-Spectrum Quotient [AQ], Multiscale Dissociation Inventory [MDI] α=0.008, Brief Illness Perception Questionnaire [BIPQ], 36-item Short-Form Health Survey [SF-36] α=0.006, Cognitive Behavioural Responses Questionnaire [CBRQ] α=0.007, Toronto Alexithymia Scale-20 [TAS-20] α=0.01). Adjusted analyses (analysis of covariance) were conducted to examine the influence of potentially confounding variables which differed significantly between groups including education, employment, ethnicity, physical health diagnoses, current treatment, symptom duration (FS/FMS/CC) and psychotropic medication (online supplemental table S18, 19).

Precipitating factors (self-reported factors occurring within 12 months before symptoms started that may have initiated disorder onset) and ongoing symptom triggers (perceived factors that make symptoms occur or get worse in the short term, on an ongoing basis) reported by the clinical groups (FS/FMS/CC), as well as BIPQ causes of illness, were independently qualitatively coded into descriptive categories (LSMM, JUJ, table 3) based on coding schemes developed by SP who then resolved any discrepancies. Functional Neurological Symptoms Questionnaire (FNSQ) data were analysed with T-tests (normal distribution) or Mann-Whitney U tests (non-normal distribution) with group (FS/FMS) as the independent variable and Cohen’s d or r (Mann-Whitney U) as measures of effect size.

Exploratory Pearson’s (normally distributed) or Spearman’s (non-normal) correlations assessing relationships between FND or depression/anxiety symptom burden and illness beliefs/perceptions, general functioning, HRQoL and cognitive-behavioural responses to symptoms, as well as clinical features and relevant aetiological factors (ie, dissociation, traumatic/adverse events, alexithymia), were run with Holm-Bonferroni corrections in cases of multiple tests on related variables.^3 4 13–17^

## Results

### Demographics

Of 252 participants screened, 200 proceeded, with 50 participants per group. Of screened FND participants, 9–19% were ineligible (mixed symptoms, ongoing severe psychiatric illness [eg, current severe alcohol or substance dependence, psychosis], extended symptom-free period) and 7–9% withdrew (travel limitations, symptom changes) ahead of their KCL visit. 82% of the FS sample and 96% of the FMS sample self-referred through charity or clinician-shared advertisements.

Participants were matched on key demographic variables including age, sex, gender and handedness (table 2). FS participants were more likely to smoke relative to HCs/CCs (difference=24–28%, ps<0.035). Higher rates of unemployment/sick leave were present in FS/FMS, significantly different to both HCs (26–34%, ps<0.001) and CCs (28–36%, ps≤0.001), and the groups differed in education, with controls completing more undergraduate and/or postgraduate courses (42–50%). The distribution of ethnicities was different in CCs compared with FS (p=0.047), and in FMS/FS compared with HCs (ps<0.004). Across the entire sample, the distribution of self-reported ethnicity was 73% white, 15% Asian, 4% black, 9% mixed ethnicity. Elevated body mass index (MD=3.29–4.67) and current medication use was reported in all clinical groups compared with HCs (ps<0.038; online supplemental table S3, S6). Current medications being taken ranged from 0 to 18 across groups, with 36–60% of FS/FMS participants taking more than four.

**Table 2:** Demographic characteristics across study groups

	FS	FMS	CC	HC	Test statistic	P value	Effect Size
Age	36.7 (13.3)	35.9 (11.1)	37.4 (13.1)	37.7 (14.9)	F (3, 196)=0.2	0.91	*η2*=0.003
BMI* (FS n=44, FMS n=49, CC n=49, HC n=49**)**	27.6 (7.4)	28.5 (8.7)	27.1 (7.3)	23.8 (4.2)	F (3, 97.9)=6.4	**<0.001**	*η2*=0.06
BMI >35 kg/m^2^	6 (12%)	9 (18%)	8 (16%)	1 (2%)	NA	**0.04†**	*v*=0.15
CGI-S*	5.0 (0.9)	4.7 (1.0)	3.60 (1.1)	NA	F (2, 97.5)=26.1	**<0.001**	*η2*=0.28
Age at onset in years	28.4 (12.6)	31.1 (12.3)	22.1 (10.3)	NA	F (2, 147)=7.7	**0.001**	*η2*=0.09
Symptom duration (years)*	8.0 (9.5)	4.8 (5.3)	15.3 (11.8)	NA	F(2, 87.4)=17.0	**<0.001**	*η2*=0.19
Sex (% female)	42 (84%)	42 (84%)	43 (86%)	41 (82%)	*χ2*=0.30	0.96	*v*=0.04
Gender (% woman)	39 (78%)	41 (82%)	42 (84%)	41 (82%)	NA	0.81†	*v*=0.10
Handedness (% right)	40 (80%)	45 (90%)	42 (84%)	46 (92%)	NA	0.12†	*v*=0.16
Smoking status (% yes)	17 (34%)	10 (20%)	5 (10%)	3 (6%)	NA	**0.001†**	*v*=0.28
Relationship (% married/cohabiting)	18 (36%)	22 (44%)	21 (42%)	18 (36%)	NA	0.93†	*v*=0.09
Dependents (% yes)	12 (24%)	11 (22%)	6 (12%)	9 (18%)	*χ2*=2.73	0.44	*v*=0.12
Carer status (% yes)	6 (12%)	3 (6%)	4 (8%)	5 (10%)	NA	0.84†	*v*=0.08
Education					*χ2*=24.20	**0.004**	*v*=0.20
Compulsory	9 (18%)	7 (14%)	3 (6%)	2 (4%)			
A-Levels	15 (30%)	19 (38%)	8 (16%)	11 (22%)			
Undergraduate	11 (22%)	17 (34%)	25 (50%)	16 (32%)			
Postgraduate	15 (30%)	7 (14%)	14 (28%)	21 (42%)			
Employment (% employed)	16 (32%)	20 (40%)	34 (68%)	33 (66%)	NA	**<0.001†**	*v*=0.28
Ethnicity (% white)	36 (72%)	45 (90%)	35 (70%)	30 (60%)	NA	**<0.001†**	*v*=0.22
Current physical health diagnosis (% yes)	32 (64%)	36 (72%)	28 (56%)	15 (30%)	*χ2*=20.14	**<0.001**	*v*=0.32
Current mental health diagnosis (% yes)	42 (84%)	34 (68%)	50 (100%)	0 (0%)	NA	**<0.001†**	*v*=0.79
Current medication (% yes)	47 (94%)	44 (88%)	40 (80%)	22 (44%)	NA	**<0.001†**	*v*=0.46
Current psychotropic medication					NA	**<0.001†**	*v*=0.38
None	11 (22%)	12 (24%)	22 (44%)	50 (100%)			
Low	5 (10%)	3 (6%)	0 (0%)	0 (0%)			
Moderate	29 (58%)	29 (58%)	27 (54%)	0 (0%)			
High	5 (10%)	6 (12%)	1 (2%)	0 (0%)			
Other treatment (% yes)	7 (14%)	21 (42%)	11 (22%)	2 (4%)	NA	**<0.001†**	*v*=0.35
Neurological family history (% yes)	29 (58%)	25 (50%)	22 (44%)	13 (26%)	*χ2*=11.88	**0.01**	*v*=0.25
Psychiatric family history (% yes)	31 (62%)	22 (44%)	35 (70%)	15 (30%)	*χ2*=20.51	**<0.001**	*v*=0.32
Other family history (% yes)	39 (78%)	41 (82%)	34 (68%)	28 (56%)	*χ2*=11.19	**0.01**	*v*=0.24

Data are mean (SD) or n (%).

Psychotropic medication defined as any medications that may alter brain functioning (eg, pregabalin, propranolol, amitriptyline).

Bold values are p<.05

* Welch’s Analysis of Variance (ANOVA).

† Fisher’s exact test.

BMI, body mass index; CC, clinical control; CGI-S, Clinical Global Impression-Severity; FMS, functional motor symptoms; FS, functional seizures; HC, healthy control.

Current self-reported physical health diagnoses were more frequent in both FND groups compared with HCs (34–42%, ps<0.01; table 2, online supplemental Table S4), and the presence of a current mental health diagnosis was more frequent in FND versus HCs (68–84%, ps<0.001) and in CCs versus all other groups (16–100%, p<0.012; online supplemental Table S5). CCs reported longer symptom duration (MD=7.3–10.6; ps<0.003) and earlier age at onset (MD=6.3–9.0; ps<0.019) relative to FS/FMS, although FND participants were rated as more clinically severe (CGI-S) on study entry (MD=1.12–1.38; ps<0.001). More FMS individuals were in some form of treatment (eg, physiotherapy/psychotherapy) compared with FS/HCs (28–38%, ps<0.017; online supplemental Table S6). There were higher rates of a family history of neurological disorder in FS (32%, p=0.007) and of psychiatric disorder in CCs/FS compared with HCs (32–40%, ps<0.001; online supplemental Table S6).

### FND symptoms

Both FND groups were matched across the number, frequency, severity and impact of FND symptoms and length of time since diagnosis (online supplemental Table S7). In FS, the most common premonitory symptom type was sensations (67%, eg, pain, dizziness; online supplemental Table S9 and S10). Ictal symptoms involved body movement in 90% of participants, and in a subset of those reporting postictal symptoms, fatigue/sleep-related phenomena were most common. In FMS, a range of primary symptoms (that which was most bothering to the participant) were reported (online supplemental Table S8), most frequently weakness (30%). Symptoms were most commonly bilateral and in the legs/feet (44%).

### Clinical characteristics

**Figure 1:**
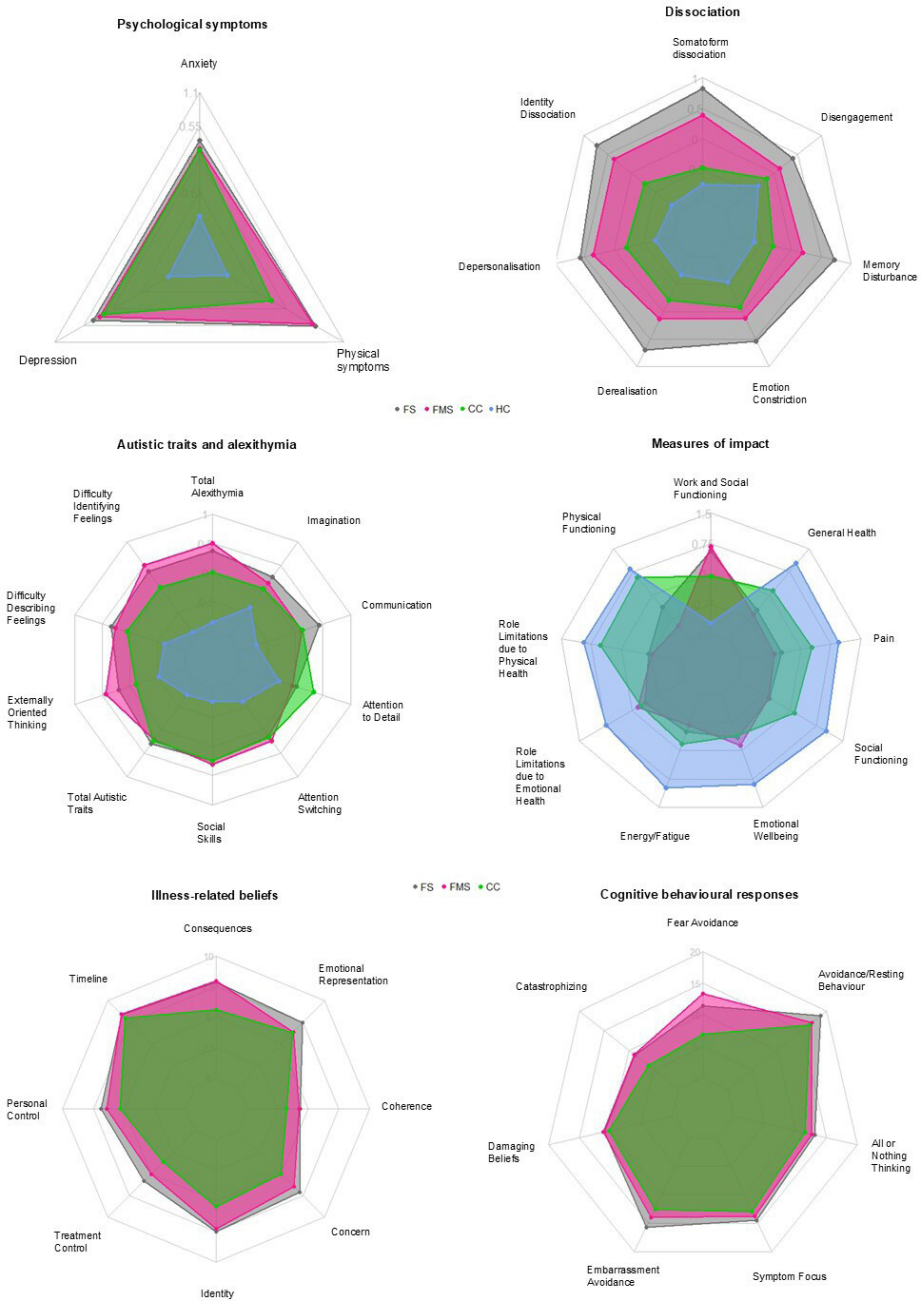
Radar plots depicting psychological stress, dissociation, alexithymia and autistic traits, health-related quality-of-life, illness beliefs and cognitive behavioural responses across participant groups. Z-scores were calculated for psychological symptoms, dissociation, illness-related beliefs and cognitive behavioural responses. CC, clinical control; FMS, functional motor symptoms; FS, functional seizures; HC, healthy control.

The clinical groups were similar in depression (Patient Health Questionnaire-9) and anxiety (Generalized Anxiety Disorder-7) severity, both significantly elevated compared with HCs (MD=6.1–9.8, online supplemental Table S9 and S10, figure 1). Increased physical symptoms (PHQ-15; MD=4.0–4.7, ps<0.001), somatoform dissociation (SDQ-20; MD=8.0–12.0, ps<0.001), disengagement (MDI; MD=11.7–19.6, ps<0.003) and depersonalisation (MDI; MD=18.9–26.0, ps<0.021) were seen in both FS and FMS compared with CCs, and in all clinical groups relative to HCs (MD=9.4–41.4, ps<0.021). In FS, derealisation and emotional constriction (MDI) were highest, significantly different from FMS and/or CCs (Derealisation: MD=15.7–23.5, ps<0.012; Emotional constriction: MD=13.2, p=0.026) and elevated in FS/FMS/CCs compared with HCs (MD=9.6–34.2, ps<0.006). The FS group also reported the highest dissociative memory disturbance (MDI) relative to all other groups (MD=18.0–42.4, ps<0.037), with elevations also reported in FMS relative to CCs/HCs (MD=15.3–24.4, ps<0.021). These results withstood sensitivity analyses (online supplemental Table S18).

Elevated autistic spectrum traits including social skills, attention switching and communication AQ subscales were seen in FS/FMS/CCs versus HCs (MD=2.0–10.0, ps<0.001; online supplemental Table S9 and S10, figure 1), with no differences among clinical groups, similarly withstanding sensitivity analyses (online supplemental Table S18). More CCs (p=0.017) and individuals with FS (p=0.012) scored above the AQ clinical cut-off (online supplemental Table S11 and S12) compared with HCs.

In FMS, overall alexithymia, difficulty identifying emotions and externally oriented thinking (TAS-20) was highest and significantly different to CCs (MD=3.0–7.7, ps<0.038), although all three clinical groups exhibited higher scores on these measures than HCs (MD=3.0–18.0, ps<0.001) and on difficulty describing feelings (MD=3.0–4.7, ps<0.011; online supplemental Table S9 and S10, figure 1), remaining significant in sensitivity analyses (online supplemental Table S18). More individuals with FS/FMS scored above the TAS-20 clinical cut-off compared with HCs (ps<0.002; online supplemental Table S11 and S12).

A higher number (MD=2.4–3.7) and impact (MD=11.0–17.9) of traumatic/adverse events was reported in FS and FMS compared with HCs (Traumatic Experiences Checklist; ps<0.008), with no significant difference to CCs (online supplemental Table S9 and S10), withstanding sensitivity analyses (online supplemental Table S18). Significantly more individuals with FS met diagnostic criteria for post-traumatic stress disorder (PTSD), scoring higher on the PDS-5 relative to all other groups (MD=12.3–23.9; ps<0.038). More individuals with FMS (p=0.001) and CCs (p=0.002) met cut-off criteria and scored higher compared with HCs (MD=7.2–11.6; online supplemental Table S11 and S12).

### Measures of impact

The three clinical groups exhibited worse HRQoL than HCs across all SF-36 subscales (MD=10.1–76.5, ps<0.033; online supplemental Table S9 and S10, figure 1). Both FND groups reported worse physical and social functioning and general health, more pain and elevated role limitations due to physical health compared with CCs (MD=15.0–57.9, ps<0.004). The FMS group reported the poorest physical functioning, significantly poorer than FS (MD=21.7, p=0.001). Reduced work and social functioning (Work and Social Adjustment Scale) was also seen in FS and FMS relative to CCs (MD=8.14–9.10, ps<0.001), and in all clinical groups compared with HCs (MD=14.9–24.0, ps<0.001). These results all remained significant in sensitivity analyses (online supplemental Table S18).

### Predisposing and precipitating factors and symptom triggers across clinical groups

In the clinical groups, self-reported precipitating factors included physical, emotional, social/relationship and environmental (table 3, online supplemental Table S13), with 42–64% of participants naming more than two factors. In FMS, physical illness/injury (eg, virus/surgery) was more frequently reported as an illness precipitant compared with CCs and FS (ps<0.044), and in FS compared with CCs (p<0.001). In FS, traumatic/adverse events (eg, bullying/sexual assault/bereavement) as well as prior mental health diagnoses (eg, depression) were more frequently cited compared with CCs (p=0.031; p=0.022).

**Table 3:** Predisposing, precipitating and perpetuating factors, symptom triggers and illness causes

	FS	FMS	CC			
Precipitating factors	n (%)	n (%)	n (%)	χ2	P value	v
Physical illness/injury	17 (34%)	28 (56%)	2 (4%)	NA	<0.001*	0.46
Work-related	16 (32%)	16 (32%)	9 (18%)	3.29	0.19	0.15
Other stressors	6 (12%)	7 (14%)	10 (20%)	1.34	0.51	0.09
Traumatic/adverse events	29 (58%)	23 (46%)	15 (30%)	7.98	**0.02**	0.23
Relationship problems	12 (24%)	6 (12%)	13 (26%)	3.50	0.17	0.15
Social/environmental	7 (14%)	10 (20%)	7 (14%)	.89	0.64	0.08
Mental health	12 (24%)	5 (10%)	2 (4%)	NA	0.02*	0.25
Other physical	3 (6%)	6 (12%)	4 (8%)	NA	0.67	0.09
Do not know, none or prefer not to say	3 (6%)	4 (8%)	9 (19%)	NA	0.18	0.17
Symptom triggers						
Physical activity	10 (20%)	24 (48%)	2 (4%)	NA	<0.001*	0.43
Emotional	23 (47%)	28 (56%)	12 (24%)	11.14	**0.004**	0.27
Mental health	9 (18%)	3 (6%)	4 (8%)	NA	0.13*	0.18
Trauma-related	6 (12%)	2 (4%)	4 (8%)	NA	0.29*	0.12
Other physical	32 (65%)	31 (62%)	13 (26%)	18.94	**<0.001**	0.36
Environmental	6 (12%)	5 (10%)	15 (30%)	8.31	**0.02**	0.24
Sensory	22 (45%)	18 (36%)	2 (4%)	NA	<0.001*	0.39
Social	8 (16%)	3 (6%)	24 (48%)	NA	<0.001*	0.42
Other psychological	12 (25%)	7 (14%)	23 (46%)	13.14	**0.001**	0.30
Other stressors	0 (0%)	0 (0%)	3 (6%)	NA	0.11*	0.20
None or do not know	4 (8%)	3 (6%)	2 (4%)	NA	0.91*	0.07
BIPQ Perceived Illness Causes†‡						
Physical symptoms/illness/injury	21 (43%)	27 (54%)	2 (4%)	NA	<0.001*	0.45
Trauma-related	24 (49%)	16 (32%)	15 (31%)	4.20	0.12	0.17
Mental health symptoms/disorder	13 (27%)	6 (12%)	2 (4%)	NA	0.01*	0.26
Genetic/familial or psychological vulnerability	8 (16%)	5 (10%)	29 (60%)	35.90	**<0.001**	0.49
Other physical	7 (14%)	20 (40%)	11 (23%)	8.86	**0.01**	0.25
Stress (general)	11 (22%)	10 (20%)	11 (23%)	.14	0.93	0.03
Childhood factors or neurodivergence	6 (12%)	2 (4%)	21 (44%)	NA	<0.001*	0.43
Work or finance-related	5 (10%)	10 (20%)	2 (4%)	NA	0.047*	0.20
Relationship dysfunction	3 (6%)	2 (4%)	6 (13%)	NA	0.26*	0.14
Environmental/social	2 (4%)	2 (4%)	8 (17%)	NA	0.046*	0.22
Other	3 (6%)	2 (4%)	7 (15%)	NA	0.17*	0.17
I do not know	7 (14%)	6 (12%)	4 (8%)	NA	0.69*	0.08
Prefer not to say or N/A	4 (8%)	8 (16%)	2 (4%)	NA	0.14*	0.17

Bold values are p<.05

* Fisher’s Exact Test.

† FS n=49.

‡ CC n=48.

BIPQ, Brief Illness Perception Questionnaire; CC, clinical control; FS, functional seizures.

Reported, perceived symptom triggers were physical, emotional, social, psychological, trauma-related, environmental and sensory (table 3, online supplemental Table S13), with more than two reported in 76–82% of participants. In FMS, physical activity (ie, exercise) and emotional triggers (ie, stress/joy) were most frequently reported (FS and/or CCs: ps<0.011), and other physical triggers (ie, hunger/pain/fatigue) were more commonly cited in both FND groups relative to CCs (ps<0.002). In FS, sensory triggers (ie, heat/lights/noise) were highest (ps<0.042), whereas social factors (ie, relationship conflict) and other psychological (ie, cognitive effort/relaxation) were more commonly reported in CCs (ps<0.002).

Perceived, self-reported causes of FS, FMS or anxiety/depression (BIPQ-Q9) covered physical health, mental health/trauma, genetics, childhood and social/environmental factors (table 3, online supplemental Table S13). In FS/FMS, more physical symptoms/illness/injury were reported as possible illness causes in relative to CCs (ps<0.001). In FS, mental health causes were more frequently cited (CC: p=0.011), while other physical factors (FS: p=0.024) were more frequently reported in FMS. In CCs, genetics/psychological vulnerability and childhood factors or neurodivergence were all more frequently disclosed relative to FS/FMS (ps<0.001).

### Illness beliefs and cognitive behavioural responses across clinical groups

Both FND groups indicated more consequences, greater impact and more concern over their illness and higher overall illness threat compared with CCs (BIPQ; MD=1.5–2.4, ps<0.017; online supplemental Table S14 and S15), figure 1), withstanding sensitivity analyses (online supplemental Table S19). Individuals with FS were more likely to believe that treatment is unlikely to help than CCs (MD=2.2, p<0.001). Both FS and FMS groups reported elevated fear avoidance and increased symptom catastrophising (CBRQ) relative to CCs (MD=2.8–6.4, p<0.001; online supplemental Table S14 and S15, figure 1). The catastrophising finding did not withstand sensitivity analyses (psychotropic medication, online supplemental Table S19).

### Exploratory correlations

In FS, greater symptom impact was associated with more illness concern (BIPQ-Concern; p=0.002; online supplemental Table S16). Role limitations due to physical health was negatively associated with fear avoidance (p=0.004), and a greater number of traumatic/adverse events was negatively related to role limitations due to emotional functioning (p<0.001). Elevated fear avoidance was also associated with poorer work and social functioning (p=0.025).

In FMS, more FND symptoms and increased severity was associated with greater perceived impact of (BIPQ-Consequences), and more symptoms associated with, their FND (BIPQ-Identity; ps<0.002; online supplemental Table S16). Greater fear avoidance was associated with more role limitations due to emotional problems (p<0.001) and fatigue (p=0.003). More traumatic/adverse events was correlated with more role limitations due to physical functioning (p=0.002).

In CCs, more traumatic/adverse events were negatively associated with physical functioning and general health (p=0.005). In all three groups, elevated depression was linked to poorer work and social functioning (ps<0.003; online supplemental Table S16).

## Discussion

This study investigated patient-reported predisposing, precipitating, perpetuating and triggering factors in FS and FMS, with an aim to understand patients’ perspectives on independent and overlapping elements of these subtypes. Participants with FS more frequently reported traumatic/adverse events and psychopathology (eg, dissociation, PTSD) as causes or precipitants of their FND and sensory symptom triggers, compared with FMS and/or CCs. In contrast, FMS participants more commonly endorsed physical illness causes or precipitating factors and physical activity and emotional symptom triggers, relative to FS and/or CCs. Both FND groups reported more physical and psychological symptoms, poorer HRQoL including general functioning and less adaptive/helpful illness-related beliefs and cognitive-behavioural responses than CCs and/or HCs. Symptom burden, illness beliefs and traumatic/adverse events were associated with different aspects of HRQoL and functioning in these FND subtypes.

**Figure 2:**
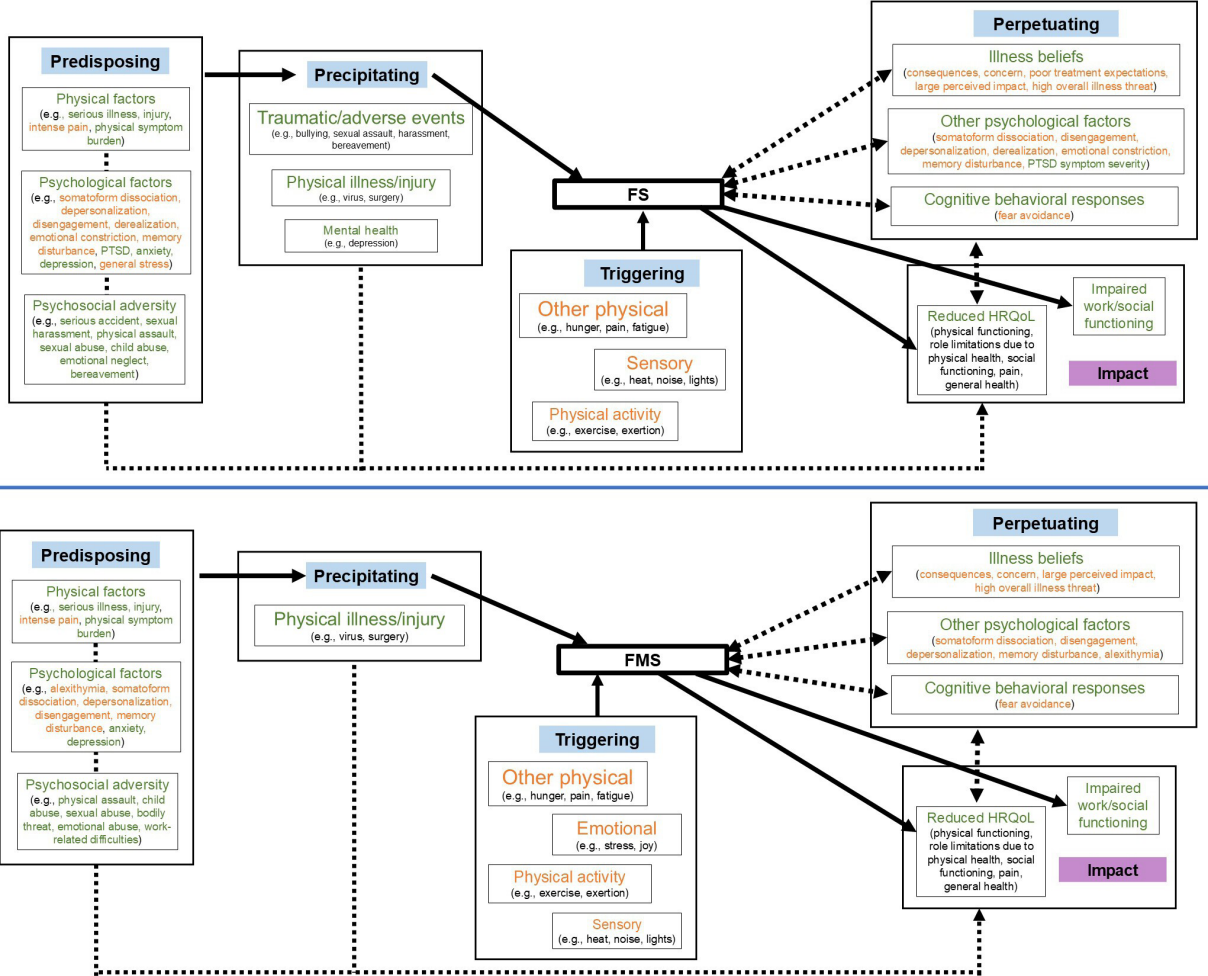
Includes changes to Millman *et al.,* (2024) with models separated into functional seizures (FS) and functional motor symptoms (FMS). These schematics represent findings in FS and FMS which were statistically significantly different from healthy and/or clinical controls. Strong evidence=green; moderate evidence=orange. Dotted arrows indicate potential relationships between variables based on limited/emerging evidence. Copyright 2024 Millman *et al*. This figure is licensed under the Creative Commons Attribution CC BY 4.0 licence. A copy of this licence is available online (https://creativecommons.org/licenses/by/4.0/). HRQoL, health-related quality of life; PTSD, post-traumatic stress disorder.

Figure 2 builds on the model presented previously,^4^ illustrating characteristics which were significantly different to both HCs and CCs in FS and FMS separately.

In FMS, physical illness/injury was the most frequently perceived, self-reported precipitating factor, significantly different to FS/CCs, suggesting the importance of considering physical factors in FMS development. This supports previous evidence indicating physical events ahead of FMS onset, where sensory information from the illness/injury may be combined with cognitive biases or panic, leading to abnormal body-focused attention and beliefs about symptoms.^20 21^ Given these findings, it may be worth routine exploration of previous physical illness or injury in the diagnostic assessment of FMS to identify possible illness-related fear and work with the patient to understand how cognitive or sensory factors may relate to their current symptoms.

In FS, traumatic/adverse events were the most commonly self-reported precipitating factor, numerically higher than FMS and significantly different to CCs, corroborating growing evidence highlighting trauma as an important risk factor in FS development.^3 16 17^ The FS group also cited mental health symptoms/disorders as possible precipitants of their illness, numerically higher than FMS and significantly different to CCs, aligning with the high presence of comorbid psychiatric disorders seen here and in other studies.^4^Although more traumatic/adverse events were reported in both FND groups relative to HCs, and symptoms of PTSD^22 23^ have been previously seen in both FS and FMS samples, significantly higher scores meeting PTSD diagnostic criteria were present in FS relative to all other groups. High rates of comorbid PTSD have been previously reported in FS^3^ and mixed samples^23^ and elevated rates of past trauma,^13^ particularly sexual and physical abuse,^3^may be important to understanding the onset and perpetuation of FS, with implications for treatment types (ie, eye movement desensitisation and reprocessing).^24^ Routine PTSD or trauma screening during diagnostic assessment may be valuable to aid in treatment planning and symptom management. Additionally, a greater number of traumatic/adverse events was significantly related to role limitations in both FND groups, though due to different types of functioning—emotional in FS and physical in FMS. These findings are consistent with the self-reported precipitating factors and impacts of the disorder on emotional and physical well-being.

Both FND groups described perceived physical causes more frequently than CCs. Distinctions between FS and FMS appeared regarding mental health, more frequent in FS relative to CCs, and other physical causes, more frequent in FMS compared with FS. These results reinforce the possible unique self-reported contributions of mental and physical health to FS and FMS.^20^ An understanding of patient’s perceived causes of their FND, particularly during assessment and in the determination of therapeutics, may be important to the collaborative formulation of a treatment plan, treatment engagement and overall clinical outcomes.

Consistent with previous studies,^4 18 25^ self-reported triggers contributing to day-to-day FND symptoms were identified by most participants and covered physical, emotional, social, psychological and environmental factors. Differences regarding physical (ie, pain/hunger; FS/FMS), sensory (FS), social (FS/CCs), physical activity (FMS) and emotional (FMS) triggers emerged as differentiators among clinical groups. Awareness of individual symptom triggers may be an important element to FND treatment, with implications for optimal therapeutic interventions, individualised approaches and a consideration of the therapeutic environment.^18^

Clinical groups were matched for depression and anxiety, suggesting that differences between them were not solely due to these variables. The FS group exhibited more derealisation (FMS/CCs), emotional constriction (CCs) and memory disturbance (FMS/CCs), building on evidence for the aetiological relevance of psychological dissociation in FND, particularly in FS.^3 10 13 14^ Due to the prominence of dissociative symptoms in FS, interventions focused on grounding and stabilisation may warrant further exploration and consideration during the development of treatment plans.

Although autistic spectrum traits were higher across clinical groups relative to HCs, there were no differences in scores or those meeting clinical cut-off criteria, suggesting that these traits may not be uniquely related to FND. All clinical groups exhibited elevated alexithymia, but FMS was the only group to score significantly higher than CCs, pointing towards the possible aetiological relevance of this trait to FMS and requiring further investigation.^15^ If replicated, a greater emphasis on emotion identification or verbalisation in the treatment of FMS could be warranted.

More threatening illness-related representations including greater consequences, concern and perceived symptom burden were reported in FS/FMS. These illness beliefs may play a perpetuating role in FND,^4 13^ further evidenced by positive associations between symptom burden and concern or consequences of the disorder. Elevated fear avoidance was similarly linked to role limitations due to physical health (FS) and emotional problems and fatigue (FMS). Early identification of these beliefs or behaviours, during diagnosis and assessment, may be of importance in guiding and tailoring treatment. Targeted interventions aimed at changing these thoughts/behaviours (ie, cognitive-behavioural therapy) may be particularly valuable to address avoidance behaviour and promote better general functioning and/or HRQoL, again found to be negatively impacted by FND in this study.^3 4 14 24 26^ The differences in how fear avoidance may relate to emotional or physical role limitations in FS and FMS suggests that tailoring cognitive-behavioural strategies to the individual/subtype would be worthwhile.

This is the largest case-control study to assess a comprehensive range of aetiological factors and symptom triggers in FS and FMS separately. Participant groups were matched across key demographic characteristics including sex, age and handedness. The CC group diagnosed with anxiety disorders and/or depression allowed for factors that may be unique to FND, and not necessarily due to elevated anxiety or depressive symptoms, to be isolated.

Although the FS and FMS samples were representative of the wider UK and FND patients more broadly due to national sampling, this may be less representative of the local area and/or patients with access to specialist services. It is possible that recruitment source may influence the illness beliefs and symptom attributions reported in this study, with self-referrers possibly more likely to view the diagnosis as central to their identity and potentially experience elevated illness coherence than those being referred by their clinician. While the exploration of FS and FMS separately is a strength of the study regarding better understanding both shared and distinct perceived aetiological factors within these subtypes, it is important to note that many people with FND experience a mixed symptom presentation. These findings may therefore not be generalisable to individuals experiencing a combination of seizures or motor symptoms. In consideration of other elements of the larger research programme,^12^ participants in the FS group were included only if they experienced warnings prior to seizure-onset. Omission of individuals without warning signs may mean this sample is less generalisable to the broader FS population, with the included sample possibly more likely to notice triggers or patterns of their symptoms. This could lead to potential biases in FS findings, with this sample possibly more vigilant towards signs or symptoms including dissociative experiences and sensory triggers.

Given the range of included self-report measures, it is possible that some scales/subscales may overlap or correlate, resulting in reduced discriminant validity (correlation matrix presented in Supplementary Materials). However, the importance of addressing potentially related but theoretically different factors and their possible roles/contributions to FS and FMS is a primary aim of this study, and all original measures were included in the reported analyses to address this. Further, medical history (eg, medications, family history) was self-reported, which may have resulted in recall bias or misreporting/omissions of relevant information. While the broader project within which this study was embedded sought to test causal and mechanistic hypotheses with other methods including experimental, neuroimaging and digital remote monitoring approaches,^12^ this particular part of the project^12^ was not designed to provide such insights. The results of the self-report measures presented here depict patients’ perspectives on what factors may have been involved in the onset or maintenance of the disorder. Lastly, it is also possible that some self-reported physical health diagnoses were incidental findings during investigations for FND. This may lead to the generation of illness labels arising from extensive testing, even in the absence of current symptoms.

These results emphasise the need to consider factors across biopsychosocial dimensions in the clinical management of people with FND. The FND groups differed in their reports of several potential aetiological factors, with FS more frequently reporting adverse life events and psychopathology as illness precipitants, in contrast to FMS more commonly endorsing physical aetiological influences. Differential symptom triggers, sensory in FS and physical and emotional in FMS, also separated these groups.

These findings suggest specific characteristics that may be involved in the development and maintenance of FS/FMS. Across subtypes, FND clearly negatively affects general functioning and HRQoL and involves specific illness-related beliefs and perceptions, which may play a perpetuating role. Individualised treatment approaches, which consider the factors that may be most relevant in these FND subtypes, might contribute to optimisation of long-term outcomes.

## Supplementary material

10.1136/jnnp-2025-337694 online supplemental file 1

## Data Availability

Data are available upon reasonable request.
