# Supplementary material for: Aetiological factors in functional seizures and functional motor symptoms: shared and distinct features
Source: J Neurol Neurosurg Psychiatry. 2026 May 7;97(8):e337694. doi: 10.1136/jnnp-2025-337694 (PMC13479279; doi:10.1136/jnnp-2025-337694)
Supplement: Supplementary file 1 [file jnnp-97-8-s001.pdf]

Supplementary Materials Table of Contents

Page 2 FND Screening Interview Guide

Page 5 Functional Neurological Symptoms Questionnaire

Page 6 Supplementary references corresponding to Table 1 within the manuscript

Page 7 Table S1. Missing data across self-report questionnaires

Page 9 Table S2. Outliers across self-report questionnaires

Page 11 Table S3. Current medications across groups

Page 12 Table S4. Current physical health diagnoses across groups

Page 13 Table S5. Current mental health diagnoses across groups

Page 14 Table S6. Significant demographics post hoc tests

Page 15 Table S7. FND symptoms by group

Page 16 Table S8. Details of FS symptoms

Page 21 Table S9. Clinical characteristics across groups

Page 23 Table S10. Significant self-report clinical measures post hoc tests

Page 26 Table S11. Proportion of participants scoring above clinical cut-offs

Page 27 Table S12. Significant clinical cut-offs post hoc tests

Page 28 Table S13. Significant precipitating factors, symptom triggers and illness causes post hoc tests

Page 29 Table S14. Illness beliefs and cognitive-behavioural responses

Page 30 Table S15. Illness beliefs post hoc tests

Page 31 Table S16. Correlations across clinical groups

Page 33 Table S17. Self-report questionnaire analyses with outliers winsorized

Page 36 Table S18. ANCOVAs for primary self-report measures across all participant groups

Page 37 Table S19. ANCOVAS for primary self-report measures across s across clinical groups (FS, FMS, CC)

Page 38 Figure S1. Correlation matrix of all included self-report measures

FND SCREENING INTERVIEW

Sociodemographic Questionnaire

PIN:

Age (years):

Sex (at birth): Male / Female / Other / Prefer not to say

Gender: Male / Female / Other / Prefer not to say

Dominant hand: Right / Left / Ambidextrous

Smoker: Y / N

Relationship status: Single / Married/Cohabiting / Separated / Divorced / Widowed

Sexuality: Straight / Gay or Lesbian / Bisexual / Other / Prefer not to say

Do you have dependents: Y / N
Details:

Are you a carer? Y / N
Details:

Highest qualification / total years of education:

Employment status (FT/PT/student/etc):

Occupation (if applicable, or occupation of highest earner in household):

Ethnicity:

Height: **Weight:** **BMI:**

Knowledge of own heart rate: Y / N

If yes, nature and estimated resting BPM:

MEDICAL HISTORY

FND HISTORY (if applicable)
*Current and previous FND symptoms (frequency, severity, impact), date of onset and diagnosis**:

CGI-S:

*FND-seizures – confirm that frequency is ≥2 / month, with premonitory symptoms
*FND-motor – confirm that symptom severity will not preclude task completion (e.g., bilateral upper limb paralysis / severe and constant tremor)

PRECIPITATING EVENTS
Were there any significant events that occurred within the 12 months before your FND symptoms started that may have triggered the onset of the disorder?

FND TRIGGERS
What do you think are the main triggers of your FND symptoms on an ongoing basis? (i.e., what makes them occur, or get worse)

PHYSICAL HEALTH HISTORY
What physical health symptoms/diagnoses do you have (other than FND)? (Ask about date of onset/diagnosis for each condition mentioned)

What physical health symptoms/diagnoses have you had in the past? (Ask about date of onset/diagnosis)

Have you ever had any significant physical injuries, accidents or major medical procedures for physical health issues? (Ask about date for each)

PSYCHIATRIC HISTORY
Have you ever been diagnosed or sought treatment for a mental health issue?

If Yes: *Have you ever received a diagnosis? (If Y, ask about frequency, severity, impact, date of onset and diagnosis)*

TREATMENTS

CURRENT

Medications:
Other:

PREVIOUS FND / PSYCHIATRIC TREATMENT

FAMILY HISTORY

Neurological (diagnoses, family member):

Psychiatric (diagnoses, family member):

Other (diagnoses, family member):

Complete Quick-SCID

ELIGIBILITY: Y / N
Reasons:

Functional Neurological Symptoms Questionnaire

Please look at the symptoms in the table below and tell us whether you have experienced these functional neurological symptoms in the **past week.** If you mark yes to indicate that the symptom was present in the past week, please complete the additional columns to tell us **how frequent** the symptoms were, **how severe** (intense) they were, and **how much impact** they had on you.

When rating the average **severity** of symptoms, please choose a number from 1 to 7, where **1=Symptom not present; 2=Minimal; 3=Mild; 4=Moderate; 5=Moderately severe; 6=Severe; 7=Very severe**. When rating the **impact** of symptoms, please choose a number from 1 to 7, where **1=No impact at all; 2=Minimal impact; 3=Mild impact; 4=Moderate impact; 5=Moderately severe impact; 6=Severe impact; 7=Very severe impact**.

**FND Symptom**	**Present?** (circle or bold)	**Frequency** (circle or bold)	**Average severity (1-7)**	**Average impact (1-7)**
Weakness	Yes / No	Constant / daily / weekly / less than weekly		
Tremor	Yes / No	Constant / daily / weekly / less than weekly		
Dystonia (muscle spasms / fixed postures)	Yes / No	Constant (1) / daily (2) / weekly (3) / less than weekly (4)		
Walking / mobility difficulties	Yes / No	Constant / daily / weekly / less than weekly		
Myoclonus (muscle jerks)	Yes / No	Constant / daily / weekly / less than weekly		
Seizures*	Yes / No	Number of seizures in the last week:		
Numbness (loss of feeling)	Yes / No	Constant / daily / weekly / less than weekly		
Visual disturbances	Yes / No	Constant / daily / weekly / less than weekly		
Sensitivity to light/sound	Yes / No	Constant / daily / weekly / less than weekly		
Dizziness	Yes / No	Constant / daily / weekly / less than weekly		
Speech / swallowing difficulties	Yes / No	Constant / daily / weekly / less than weekly		
Cognitive difficulties (e.g., brain fog, memory lapses)	Yes / No	Constant / daily / weekly / less than weekly		
Other FND symptoms	Details:	Constant / daily / weekly / less than weekly		

Please tell us which FND symptom(s) is most severe and has the most impact on you.

*If you experience FND seizures, do you have warning symptoms? Yes / No

*If you experience warning symptoms before an FND seizure, what is the earliest or most consistent symptom(s) that you experience?

As presented in Pick, David et al. 2024. This is licensed under the Creative Commons Attribution CC BY 4.0 licence. To view a copy of this licence visit https://creativecommons.org/licenses/by/4.0/.

Supplementary references corresponding to Table 1 within the manuscript

S1. Spitzer RL, Kroenke K, Williams JB, Löwe B. A brief measure for assessing generalized anxiety disorder: the GAD-7. Archives of internal medicine. 2006;166(10):1092-7.

S2. Kroenke K, Spitzer RL, Williams JB. The PHQ-9: validity of a brief depression severity measure. Journal of general internal medicine. 2001;16(9):606-13.

S3. Kroenke K, Spitzer RL, Williams JB. The PHQ-15: validity of a new measure for evaluating the severity of somatic symptoms. Psychosomatic medicine. 2002;64(2):258-66.

S4. Briere J, Weathers FW, Runtz M. Is dissociation a multidimensional construct? Data from the Multiscale Dissociation Inventory. Journal of Traumatic Stress: Official Publication of the International Society for Traumatic Stress Studies. 2005;18(3):221-31.

S5. Nijenhuis ER, Spinhoven P, Van Dyck R, Van Der Hart O, Vanderlinden J. The development and psychometric characteristics of the Somatoform Dissociation Questionnaire (SDQ-20). The Journal of nervous and mental disease. 1996;184(11):688-94.

S6. Baron-Cohen S, Wheelwright S, Skinner R, Martin J, Clubley E. The autism-spectrum quotient (AQ): evidence from Asperger syndrome/high-functioning autism, males and females, scientists and mathematicians. Journal of autism and developmental disorders. 2001;31(1):5-17.

S7. Bagby RM, Parker JD, Taylor GJ. The twenty-item Toronto Alexithymia Scale—I. Item selection and cross-validation of the factor structure. Journal of psychosomatic research. 1994;38(1):23-32.

S8. Nijenhuis ER, Van der Hart O, Kruger K. The psychometric characteristics of the Traumatic Experiences Checklist (TEC): First findings among psychiatric outpatients. Clinical Psychology & Psychotherapy: An International Journal of Theory & Practice. 2002;9(3):200-10.

S9. Foa EB, McLean CP, Zang Y, Zhong J, Powers MB, Kauffman BY, et al. Psychometric properties of the Posttraumatic Diagnostic Scale for DSM–5 (PDS–5). Psychological assessment. 2016;28(10):1166.

S10. Broadbent E, Petrie KJ, Main J, Weinman J. The brief illness perception questionnaire. Journal of psychosomatic research. 2006;60(6):631-7.

S11. Ryan EG, Vitoratou S, Goldsmith KA, Chalder T. Psychometric properties and factor structure of a long and shortened version of the cognitive and behavioural responses questionnaire. Psychosomatic medicine. 2018;80(2):230-7.

S12. Hays RD, Sherbourne CD, Mazel RM. The rand 36-item health survey 1.0. Health economics. 1993;2(3):217-27.

S13. Mundt JC, Marks IM, Shear MK, Greist JM. The Work and Social Adjustment Scale: a simple measure of impairment in functioning. The British Journal of Psychiatry. 2002;180(5):461-4.

Table S1. Missing data across self-report questionnaires

Variable	*Missing after imputation*	*% missing*
**SDQ20 (n=197)**	1	.5
**TAS20 (n=196)**	2	1
**TASdifficultidentify (n=196)**	2	1
**TASdifficultdescribe (n=196)**	2	1
**TASexternalorient (n=196)**	2	1
**AQ (n=196)**	2	1
**Aqsocialskill (n=195)**	3	1.5
**Aqattnswitch (n=196)**	2	1
**Aqattntodetail (n=196)**	2	1
**Aqcommun (n=197)**	1	.5
**Aqimagine (n=196)**	2	1
**MDID_T (n=198)**	0	0
**MDIDP_T (n=198)**	0	0
**MDIDR_T (n=197)**	1	.5
**MDIEC_T (n=198)**	0	0
**MDIMD_T (n=198)**	0	0
**MDIID_T (n=198)**	0	0
**GAD7 (n=195)**	3	1.5
**PHQ9 (n=197)**	1	.5
**WSAS (n=198)**	0	0
**PHQ15 (n=198)**	0	0
**SF36_PHYSF (n=198)**	0	0
**SF36_RL_PHYS (n=197)**	1	.5
**SF36_RL_EMOT (n=197)**	1	.5
**SF36_ENERGY (n=188)**	10	5
**SF36_EMOTWB (n=198)**	0	0
**SF36_SOCF (n=196)**	2	1
**SF36_PAIN (n=198)**	0	0
**SF36_GENHEALTH (n=198)**	0	0
**TEC_total (n=198)**	0	0
**TEC_impact (n=198)**	0	0
**PDS5 (n=194)**	4	2
**BIPQ_consequences (n=147)**	1	.6
**BIPQ_timeline (n=142)**	6	4
**BIPQ_personalctl (n=147)**	1	.6
**BIPQ_treatmentctl (n=133)**	15	10
**BIPQ_identity (n=147)**	1	.6
**BIPQ_concern (n=147)**	1	.6
**BIPQ_coherence (n=147)**	1	.6
**BIPQ_emotrep (n=147)**	1	.6
**BIPQ_illnessthreat (n=142)**	6	4

**CBRQ_FearAvoid (n=147)**	1	.6
**CBRQ_Catastrophizing (n=146)**	2	1.4
**CBRQ_DamagingBeliefs (n=147)**	1	.6
**CBRQ_EmbarrassmentAvoidance (n=147)**	1	.6
**CBRQ_SymptomFocus (n=147)**	1	.6
**CBRQ_AllorNothing (n=147)**	1	.6
**CBRQ_AvoidanceResting (n=147)**	1	.6
**SymptomCount (n=100)**	0	0
**SymptomFrequency (n=100)**	0	0
**SymptomSeverity (n=100)**	0	0
**SymptomImpact (n=100)**	0	0

Notes. In cases where missing data on a given measure exceeded 20%, the participant was excluded from that analysis. Where missing data did not exceed 20% on a given measure, the missing items were imputed with the mean of that individual's score on the scale/subscale.

Table S2. Outliers across self-report questionnaires

Variable	*n*	%
**SDQ20 (n=197)**	4	2
**TAS20 (n=196)**	1	.5
**TASdifficultidentify (n=196)**	1	.5
**TASdifficultdescribe (n=196)**	1	.5
**TASexternalorient (n=196)**	3	1.5
**AQ (n=196)**	1	.5
**Aqsocialskill (n=195)**	1	.5
**Aqattnswitch (n=196)**	0	0
**Aqattntodetail (n=196)**	0	0
**Aqcommun (n=197)**	2	1
**Aqimagine (n=196)**	0	0
**MDID_T (n=198)**	1	.5
**MDIDP_T (n=198)**	3	1.5
**MDIDR_T (n=197)**	3	1.5
**MDIEC_T (n=198)**	5	2.5
**MDIMD_T (n=198)**	3	1.5
**MDIID_T (n=198)**	10	5
**GAD7 (n=195)**	2	1
**PHQ9 (n=197)**	1	.5
**WSAS (n=198)**	1	.5
**PHQ15 (n=198)**	1	.5
**SF36_PHYSF (n=198)**	3	1.5
**SF36_RL_PHYS (n=197)**	7	4
**SF36_RL_EMOT (n=197)**	3	1.5
**SF36_ENERGY (n=188)**	1	.5
**SF36_EMOTWB (n=198)**	0	0
**SF36_SOCF (n=196)**	1	.5
**SF36_PAIN (n=198)**	2	1
**SF36_GENHEALTH (n=198)**	3	1.5
**TEC_total (n=198)**	3	1.5
**TEC_impact (n=198)**	6	3
**PDS5 (n=194)**	3	1.5
**BIPQ_consequences (n=147)**	2	1
**BIPQ_timeline (n=142)**	2	1
**BIPQ_personalctl (n=147)**	0	0
**BIPQ_treatmentctl (n=133)**	0	0
**BIPQ_identity (n=147)**	1	1
**BIPQ_concern (n=147)**	1	1
**BIPQ_coherence (n=147)**	1	1
**BIPQ_emotrep (n=147)**	0	0
**BIPQ_illnessthreat (n=142)**	0	0
**CBRQ_FearAvoid (n=147)**	0	0

**CBRQ_Catastrophizing (n=146)**	0	0
**CBRQ_DamagingBeliefs (n=147)**	0	0
**CBRQ_EmbarrassmentAvoidance (n=147)**	0	0
**CBRQ_SymptomFocus (n=147)**	2	1
**CBRQ_AllorNothing (n=147)**	3	2
**CBRQ_AvoidanceResting (n=147)**	0	0
**SymptomCount (n=100)**	0	0
**SymptomFrequency (n=100)**	2	2
**SymptomSeverity (n=100)**	2	2
**SymptomImpact (n=100)**	2	2

Notes. Outliers = ±2.5 standard deviations from the mean on a given variable.

Table S3. Current medications across groups

	FS	FMS	CC	HC
**Drug type/class**	***n (%)***	***n*** (%)	***n*** (%)	***n*** (%)
Anticonvulsants	17 (34%)	15 (30%)	3 (6%)	0 (%)
Antihistamines	9 (18%)	12 (24%)	6 (12%)	0 (%)
Anti-infectives, antivirals or immunosuppressants	2 (4%)	5 (10%)	0 (%)	0 (%)
Benzodiazepines	5 (10%)	2 (4%)	3 (6%)	0 (%)
Diabetes medications	0 (%)	3 (6%)	0 (%)	0 (%)
Dopaminergic and anticholinergic agents	3 (6%)	2 (4%)	0 (%)	0 (%)
Cardiovascular or hematologic medications	19 (38%)	25 (50%)	15 (30%)	5 (10%)
Central nervous system stimulants	4 (8%)	0 (%)	2 (4%)	0 (%)
Contraception	6 (12%)	9 (18%)	8 (16%)	9 (18%)
Corticosteroids or analgesics including nonsteroidal anti-inflammatories	13 (26%)	13 (26%)	4 (8%)	3 (6%)
Gastrointestinal medications	13 (26%)	29 (58%)	3 (6%)	0 (%)
Hormones/hormone replacement therapy	6 (12%)	5 (10%)	11 (22%)	8 (16%)
Muscle relaxants	3 (6%)	2 (4%)	2 (4%)	0 (%)
Opiates	5 (10%)	13 (26%)	3 (6%)	0 (%)
Other psychiatric medications (e.g., antipsychotics, antimanic agents, monoamine oxidase inhibitors)	5 (10%)	3 (6%)	3 (6%)	0 (%)
Respiratory medications	11 (22%)	11 (22%)	5 (10%)	3 (6%)
Antidepressants (e.g., selective serotonin reuptake inhibitors; tetracyclic or tricyclics)	44 (88%)	42 (84%)	26 (52%)	0 (%)
Urologic medications	0 (%)	2 (4%)	0 (%)	0 (%)

Table S4. Current physical health diagnoses across groups.

	FS	FMS	CC	HC
**Physical health disorder**	***n (%)***	***n*** (%)	***n*** (%)	***n*** (%)
Respiratory/breathing-related disorders	12 (24%)	10 (20%)	9 (18%)	3 (6%)
Allergies and skin conditions	4 (8%)	5 (10%)	3 (6%)	0 (0%)
Musculoskeletal disorders	5 (10%)	9 (18%)	5 (10%)	5 (10%)
Cardiovascular, circulatory or blood disorders and/or reproductive disorders	12 (24%)	15 (30%)	11 (22%)	3 (6%)
Functional somatic syndromes (e.g., fibromyalgia, IBS, CFS)	13 (26%)	12 (24%)	2 (4%)	0 (0%)
Hypermobility	3 (6%)	12 (24%)	3 (6%)	0 (0%)
Gastrointestinal diseases or urinary system disorders	3 (6%)	5 (10%)	4 (8%)	2 (4%)
Metabolic disorders	7 (14%)	7 (14%)	4 (8%)	2 (4%)
Immune-related disorders	0 (0%)	2 (4%)	0 (0%)	0 (0%)
Long covid	2 (4%)	2 (4%)	0 (0%)	0 (0%)
Neurological disorders (e.g., migraine/headache disorders, chiari malformation, Tourette's, cerebral small vessel disease)	10 (20%)	10 (20%)	2 (4%)	0 (0%)
Sensory disorders (e.g., hearing loss, glaucoma, Meniere's)	3 (6%)	2 (4%)	2 (4%)	0 (0%)

Table S5. Mental health diagnoses across groups

	FS	FMS	CC	HC
**Quick-SCID diagnoses**	***n (%)***	***n (%)***	***n (%)***	***n (%)***
Major Depressive Disorder	33 (66%)	34 (68%)	48 (96%)	0 (0%)
Bipolar I	2 (4%)	3 (6%)	3 (6%)	0 (0%)
Bipolar II	3 (6%)	0 (0%)	0 (0%)	0 (0%)
12-Month Non-Alcohol Substance Use Disorder	0 (0%)	0 (0%)	0 (0%)	0 (0%)
Current Panic Disorder	5 (10%)	3 (6%)	3 (6%)	0 (0%)
Current Agoraphobia	6 (12%)	4 (8%)	4 (8%)	0 (0%)
Current Social Anxiety Disorder	4 (8%)	4 (8%)	8 (16%)	0 (0%)
Current Generalized Anxiety Disorder	11 (22%)	9 (18%)	22 (44%)	0 (0%)
**Self-report current diagnoses**				
Anxiety disorders	34 (68%)	30 (60%)	50 (100%)	0 (0%)
Mood disorders	35 (70%)	30 (60%)	47 (94%)	0 (0%)
Eating disorders	3 (6%)	3 (6%)	2 (4%)	0 (0%)
Trauma-related disorders	14 28%)	8 (16%)	2 (4%)	0 (0%)
Neurodevelopmental disorders	10 (20%)	2 (4%)	4 (8%)	0 (0%)
Personality disorders	5 (10%)	4 (8%)	3 (6%)	0 (0%)
Other: Dissociative Disorders, Obsessive Compulsive Disorder	4 (8%)	3 (6%)	3 (6%)	0 (0%)

Table S6. Significant demographics post hoc tests

Variable	Group comparison	MD	95% CI	p
**Body Mass Index***	CC - HC	3.29	[6.46, .13]	.038
	FMS - HC	4.67	[8.28, 1.05]	.006
	FS - HC	3.82	[7.11, .53]	.017
**Clinical Global Impression-Severity***	CC - FMS	-1.12	[-.63, -1.61]	<.001
	CC - FS	-1.38	[-.91, -1.85]	<.001
**Age of onset***	CC - FMS	-8.98	[-3.57, -14.4]	<.001
	CC - FS	-6.32	[-.85, -11.8]	.019
**Symptom duration**	CC - FMS	10.6	[14.9, 6.17]	<.001
	CC - FS	7.32	[12.4, 2.22]	.003
**Smoking status^**	CC - FS	NA	NA	.035
	FS - HC	NA	NA	.005
**Education^**	FMS – HC	NA	NA	.042
**Employment^**	CC - FMS	NA	NA	.001
	CC - FS	NA	NA	<.001
	FMS - HC	NA	NA	<.001
	FS - HC	NA	NA	<.001
**Ethnicity^**	CC - FS	NA	NA	.047
	FMS - HC	NA	NA	.004
	FS - HC	NA	NA	.008
**Current physical health^**	FMS - HC	NA	NA	<.001
	FS - HC	NA	NA	.01
**Current mental health^**	CC - FMS	NA	NA	<.001
	CC - FS	NA	NA	.012
	CC - HC	NA	NA	<.001
	FMS - HC	NA	NA	<.001
	FS - HC	NA	NA	<.001
**Current medication^**	CC - HC	NA	NA	.002
	FMS - HC	NA	NA	<.001
	FS - HC	NA	NA	<.001
**Current psychotropic medication^**	CC – FMS	NA	NA	.037
	CC – FS	NA	NA	.020
	CC - HC	NA	NA	<.001
	FMS - HC	NA	NA	<.001
	FS - HC	NA	NA	<.001
**Current other treatment^**	FS - FMS	NA	NA	.017
	FMS - HC	NA	NA	<.001
**Neuro fam history^**	FS - HC	NA	NA	.007
**Psych fam history^**	CC - HC	NA	NA	.001
	FS - HC	NA	NA	.009

Notes. *Games-Howell; ^Pairwise with Holm correction.

Table S7. FND symptoms by group.

Variable	FS		FMS
	**n (%)**		**n (%)**
**Premonitory symptoms (n=49)**		**Primary symptom**	
Consciousness	19 (39%)	*Dystonia*	5 (10%)
Movement	18 (37%)	*Weakness*	15 (30%)
Emotions	2 (4%)	*Tics or stuttering*	2 (4%)
Sensations	33 (67%)	*Myoclonus*	6 (12%)
Cognition	16 (33%)	*Gait*	8 (16%)
Speech/communication	13 (27%)	*Paralysis*	5 (10%)
Cardiovascular	4 (8%)	*Tremor*	9 (18%)
Gastrointestinal	4 (8%)	*Laterality*	
Other autonomic	2 (4%)	*Right-sided*	9 (18%)
Fatigue/sleep-related	8 (16%)	*Left-sided*	9 (18%)
**Ictal symptoms**		*Bilateral*	27 (54%)
Consciousness	29 (58%)	*Bilateral (worse on right)*	3 (6%)
Movement	45 (90%)	*Bilateral (worse on left)*	2 (4%)
Emotions	2 (4%)	**Affected body parts**	
Sensations	6 (12%)	*Whole body*	12 (24%)
Speech/communication	21 (42%)	*Face*	4 (8%)
Cardiovascular	0 (0%)	*Legs/feet*	22 (44%)
Gastrointestinal	2 (4%)	*Hands/arms*	3 (6%)
Other autonomic	0 (0%)	*Multiple*	3 (6%)
Fatigue/sleep-related	3 (6%)	*Limbs (legs and arms)*	6 (12%)
**Post-ictal symptoms (n=28)**			
Consciousness	0 (0%)		
Movement	5 (18%)		
Emotions	4 (14%)		
Sensations	7 (25%)		
Cognition	7 (25%)		
Speech/communication	4 (14%)		
Cardiovascular	0 (0%)		
Gastrointestinal	0 (0%)		
Fatigue/sleep-related	18 (64%)		
	**FS**	**FMS**	
**Variable**	**M (SD)**<br>**Mdn (IQR)**	**M (SD)**<br>**Mdn (IQR)**	
**Symptom Count**	7.4 (3.3)<br>7.0 (5.0)	7.6 (2.6)<br>8.0 (4.5)	t=.37 (98), p=.71, d=.08
**Symptom Frequency**	23.6 (17.6)<br>19.0 (19.5)	21.7 (9.59)<br>22.5 (13.0)	W=1273, p=.88, r=.02
**Average Severity**	4.2 (1.1)<br>4.1 (1.1)	4.2 (1.0)<br>4.3 (.9)	W=1337, p=.55, r=.06
**Average Impact**	4.1 (1.2)<br>4.1 (.9)	4.2 (1.1)<br>4.2 (1.1)	W=1370, p=.41, r=.08
**Time since diagnosis**	2.6 (3.9)<br>1.3 (2.8)	2.6 (2.1)<br>2.0 (2.0)	W=1420, p=.12, r=.16

Table S8. Details of premonitory, ictal and post-ictal symptoms in functional seizures.

Symptom Type	Seizure Symptoms	Premonitory Symptoms	Post-ictal symptoms
**Consciousness**	Loss of awareness	In and out of awareness	
	Full loss of consciousness/absences	Doesn't know where they are	
	Altered awareness - absences	Disorientation	
	Unresponsive - loss of control/strength	Spaced out	
	Feel like brain is "losing its grip" on the world or as if in a tunnel	Reduced responsiveness/awareness	
	Black out/loss of awareness	Staring off into distance	
	Trapped inside self/out of body experience	Feeling disconnected	
	Absences/blankness - altered awareness, unresponsive	Dissociation	
	Vacant	Vacant stare	
	Unresponsive	Out of body	
	Out-of-body experience	Go blank	
	Unresponsive / like a dream	Similar to concussion, things get fuzzy	
	Feels faint	Feeling like rising out of body	
		Sometimes similar to being drunk	
		Feeling wobbly, like going to float away	
		Feeling faint	
		Going distant	
		Rising feeling	
**Movements**	Major motor symptoms	Jaw slips	Limb twitches
	Completely locked in	Arm/face spasms	Feet curl up
	Tonic-clonic, body tremors	Eyes rolling back	Weakness
	Motor symptoms	Eyes lock to the right	Difficulty moving
	Can't hold up, can't move, collapse / tonic-clonic, hands can claw up, eyes can flicker	Arm moves to the right	
	Minor-major motor symptoms	Close eyes and then it happens	

	Convulsions	Eyes flickering	
	Minor motor symptoms	Lean to left and eyes start twitching	
	Drops/falls	Involuntary clicking sound with tongue	
	Can't move	Discoordination	
	Can't open eyes, left arm and leg can't move	Weakness	
	Weakness/limp	Facial tics/spasms	
	Shaking	Wobbly	
	Eyes space out/close	Loss of movement	
	Stiffness	Left eye twitches	
		Head backwards or forwards first	
		Upper body shaking	
		Twitches	
		Unable to move	
		Neck jolts back	
**Emotions**	Crying	Panic	Crying
		Very emotional	Lack of motivation
**Sensations**	Pain	Neck pain	Whole body aches
	Severe head pain	Start with sharp pain	Muscle pain
	Glitter tunnels - glitter in vision	Dots in vision or cloudy	Numbness in left side
	Whole body feels like it's spinning	Headache	Dizziness
	Double vision	Creeping feeling on back of neck	Headache
		Ringing in ears	Pain
		Pain above ears and in neck	Metallic taste in mouth
		Upper body starts feeling disconnected	
		Ears block up	
		Depth perception is off	
		Headache	
		Dizzy	

		Pressure in different parts of head	
		Really debilitating headache	
		Visual disturbance	
		Unusual feeling in belly/head	
		Feeling cold/freezing down the whole spine	
		Intense 'itching' in middle of brain	
		Sharp pain on right side of head	
		Lightheaded	
		Blurry vision	
		Pressure in head	
		Nauseous headache	
		Pressure behind both eyes	
		Body feels weird	
		Eyes 'melting'	
		Senses shut down	
		Eyes out of focus	
		Warning is visual, like a migraine	
		Pressure	
		Floor feels like it's sinking	
		Dots in vision	
		Right shoulder tension	
**Cognition**	Memory loss	Trouble concentrating	Disoriented
		Forget what saying	Memory loss
		Feels ‘funny’	Brain fog
		Can feel it coming	Memory impacted
		Weird feeling	No memory for seizures
		Aura	Confusion
		Hard to concentrate	Confusion/cognitive symptoms

		Brain fog	
		Difficulty finding words	
		Fogginess	
		Confused	
		Sometimes feel a bit weird	
		Just know when things are going to happen	
		Become very forgetful	
		Brain slowed down	
		Deja vu	
**Speech**	Cannot communicate	Speech difficulties	Speech impacted
	Vocalizations	Speech goes	Difficulty talking
	Words won't come out	Slurred speech	
	Non-verbal vocalizations	Vocal tics	
	Struggles to talk	Can't speak	
	Stutter	Stuttering, stop speaking	
	Lose ability to speak	Speech slows	
	Speech slurred	Stuttering	
	Speech impairment		
	Can't respond/communicate		
	Can hear but can't talk		
**Fatigue/sleep-related**	Dead weight	Feel lethargic	Fatigue
	Heavy eyes	Sudden fatigue	Exhaustion
	Fall asleep	Eyes will feel very heavy	Tiredness
		Limb heaviness	
		Need to lie down	
		Tired really quickly	
		Very heavy head	
**Gastrointestinal**	Stomach pain	Rising in stomach	

	Vomiting	Rising feeling in stomach	
		Feeling sick	
**Cardiovascular**		Blood pressure drops	
		Sometimes can feel it coming like a pressure in the chest	
		Heart palpitations	
		Heavy chest	
**Other autonomic**		Hot/sweaty/clammy	Sweating
		Dry mouth	

Table S9. Clinical characteristics.

	FS	FMS	CC	HC			
Variable	M (*SD*)<br>Mdn (IQR)	M (*SD*)<br>Mdn (IQR)	M (*SD*)<br>Mdn (IQR)	M (*SD*)<br>Mdn (IQR)		*p*	
**Generalized Anxiety disorder-7* (FS n=47)**	9.9 (6.4)<br>8.0 (11.0)	8.6 (6.0)<br>7.0 (10.8)	8.9 (5.1)<br>8.0 (6.3)	2.5 (3.0)<br>1.0 (4.0)	F=34.3 (3, 99.8)	**<.001**	*η2*=.24
**Patient Health Questionnaire-9* (FS n=49)**	12.3 (6.8)<br>11.0 (8.0)	11.0 (6.5)<br>9.0 (9.8)	10.9 (6.7)<br>10.0 (11.2)	2.5 (2.4)<br>2.0 (3.8)	F=63.6 (3, 94.0)	**<.001**	*η2*=.31
**Patient Health Questionnaire-15***	14.9 (6.1)<br>14.0 (8.0)	14.2 (4.5)<br>15.0 (5.8)	10.2 (4.8)<br>10.0 (7.0)	4.8 (3.2)<br>4.0 (4.5)	F=65.5 (3, 105.0)	**<.001**	*η2*=.42
**Posttraumatic Diagnostic Scale-5* (FS n=48, FMS n=49)**	27.4 (24.5)<br>30.5 (47.0)	15.1 (19.3)<br>7.5 (27.0)	10.7 (15.7)<br>0.0 (20.8)	3.5 (5.9)<br>0.0 (5.5)	F=20.0 (3, 89.5)	**<.001**	*η2*=.20
**Somatoform Dissociation Questionnaire-20* (FS n=49)**	35.0 (11.1)<br>32.0 (11.0)	31.1 (7.1)<br>30.5 (9.5)	23.0 (5.1)<br>21.0 (3.0)	20.6 (1.2)<br>20.0 (1.0)	F=62.7 (3, 84.4)	**<.001**	*η2*=.41
**Traumatic Experiences Checklist total***	7.5 (5.9)<br>6.0 (9.8)	6.2 (3.6)<br>6.0 (5.8)	5.2 (3.9)<br>5.0 (4.3)	3.8 (3.7)<br>3.0 (4.8)	F=6.0 (3, 107.0)	**.001**	*η2*=.09
**Traumatic Experiences Checklist impact***	30.3 (27.0)<br>20.5 (41.0)	23.4 (15.1)<br>21.0 (22.8)	19.0 (15.3)<br>16.0 (22.0)	12.4 (14.8)<br>8.0 (14.0)	F=7.5 (3, 106.0)	**<.001**	*η2*=.11
**Multiscale Dissociation Inventory**							
**Disengagement***	84.6 (18.0)<br>84.0 (27.8)	76.7 (18.9)<br>76.0 (20.0)	65.0 (12.8)<br>64.0 (21.0)	53.9 (9.5)<br>52.0 (12.0)	F=47.6 (3, 104.0)	**<.001**	*η2*=.37
**Depersonalization***	89.5 (39.6)<br>82.0 (53.0)	82.3 (37.4)<br>74.0 (53.0)	63.4 (24.8)<br>51.5 (27.0)	48.1 (5.2)<br>47.0 (0.0)	F=35.4 (3, 84.5)	**<.001**	*η2*=.23
**Derealization* (FS n=49)**	81.2 (27.9)<br>74.0 (33.0)	65.6 (21.1)<br>60.0 (31.8)	57.8 (20.8)<br>52.0 (17.0)	47.1 (2.5)<br>46.0 (0.0)	F=39.7 (3, 81.9)	**<.001**	*η2*=.28
**Emotional Constriction***	71.6 (26.7)<br>64.0 (35.0)	62.7 (22.5)<br>50.0 (30.0)	58.4 (18.4)<br>50.0 (18.0)	48.8 (6.5)<br>46.0 (0.0)	F=18.5 (3, 91.5)	**<.001**	*η2*=.15
**Memory Disturbance***	93.3 (36.2)<br>83.0 (56.0)	75.3 (29.4)<br>64.0 (38.0)	60.0 (21.5)<br>53.0 (21.5)	50.9 (11.1)<br>45.0 (7.0)	F=28.1 (3, 97.2)	**<.001**	*η2*=.28
**Identity Dissociation*”**	66.5 (39.7)<br>47.0 (12.0)	59.3 (31.5)<br>47.0 (0.0)	52.0 (13.4)<br>47.0 (0.0)	47 (0.0)<br>47 (0.0)	F=3.6 (2, 80.7)	.031	*η2*=.04
**Autism Spectrum Quotient**							
**Total* (FS n=49, HC n=49)**	24.8 (10.3)<br>23.0 (13.0)	23.9 (9.6)<br>23.0 (12.0)	24.2 (9.5)<br>24.0 (13.0)	14.8 (6.9)<br>14.0 (10.0)	F=18.2 (3, 105.0)	**<.001**	*η2*=.17
**Social Skills* (FS n=49, FMS n=49, HC n=49)**	4.8 (3.0)<br>5.0 (4.0)	4.9 (2.6)<br>5.0 (4.0)	4.8 (2.7)<br>5.0 (4.3)	2.0 (2.0)<br>1.0 (3.0)	F=20.7 (3, 105.0)	**<.001**	*η2*=.19
**Attention Switching (FS n=48)**	6.3 (2.7)<br>6.5 (3.0)	6.4 (2.3)<br>6.0 (3.8)	6.2 (2.8)<br>7.0 (4.0)	4.2 (2.1)<br>4.0 (2.0)	F=8.7 (3, 192)	**<.001**	*η2*=.12
**Attention to Detail (FS n=49, HC n=49)**	5.3 (2.4)<br>5.0 (3.0)	5.2 (2.3)<br>5.0 (3.0)	6.0 (2.3)<br>5.5 (4.0)	4.6 (2.2)<br>5.0 (3.0)	F=2.7 (3, 192)	.048	*η2*=.04
**Communication* (FS n=49)**	4.8 (2.9)<br>5.0 (4.0)	3.9 (2.6)<br>3.0 (4.0)	3.9 (2.9)<br>3.0 (4.0)	1.6 (1.9)<br>1.0 (2.0)	F=18.4 (3, 105.0)	**<.001**	*η2*=.17
**Imagination (FS n=49, HC n=49)**	3.9 (3.1)<br>3.0 (3.0)	3.6 (2.6)<br>3.0 (3.0)	3.3 (2.1)<br>3.0 (2.0)	2.3 (1.9)<br>2.0 (3.0)	F=3.9 (3, 192)	.01	*η2*=.06
**Toronto Alexithymia Scale-20**							
**Total (FS n=49, HC n=49)**	55.6 (12.8)<br>57.0 (18.0)	57.1 (13.4)<br>58.0 (20.8)	49.4 (12.0)<br>47.5 (19.2)	39.1 (11.0)<br>37.0 (11.0)	F=21.5 (3, 192)	**<.001**	*η2*=.25
**Difficulty Identifying**	20.8 (6.1)<br>22.0 (6.0)	21.6 (6.6)<br>21.0 (11.8)	18.2 (5.8)<br>18.5 (10.0)	12.4 (5.0)<br>11.0 (6.0)	F=24.5 (3, 192)	**<.001**	*η2*=.28

**Emotions (FS n=49, HC n=49)**							
**Difficulty Describing Emotions (FS n=49, HC n=49)**	15.4 (4.8)<br>17.0 (6.0)	14.9 (5.2)<br>16.0 (8.8)	13.8 (4.8)<br>13.5 (7.0)	10.7 (4.6)<br>10.0 (7.0)	F=9.2 (3, 192)	**<.001**	*η2*=.13
**Externally Oriented Thinking**	19.4 (5.2)<br>20.0 (7.0)	20.5 (4.5)<br>21.0 (6.8)	17.5 (4.8)<br>17.0 (6.0)	16.0 (5.1)<br>16.0 (7.0)	F=8.4 (3, 192)	**<.001**	*η2*=.12
**36-Item Short Form Health Survey**							
**Physical Functioning* (FS n=49)**	55.0 (29.1)<br>55.0 (50.0)	33.3 (27.1)<br>25.0 (38.8)	85.8 (22.6)<br>97.5 (16.2)	95.9 (6.1)<br>100.0 (5.0)	F=111.0 (3, 87.5)	**<.001**	*η2*=.55
**Role Limitations due to Physical Health* (FS n=49)**	16.3 (30.0)<br>0.0 (25.0)	14.0 (25.8)<br>0.0 (25.0)	71.9 (39.8)<br>100.0 (50.0)	90.5 (24.2)<br>100.0 (0.0)	F=103.0 (3, 105.0)	**<.001**	*η2*=.56
**Role Limitations due to Emotional Health* (FMS n=48)**	38.0 (44.7)<br>0.0 (100.0)	50.7 (45.6)<br>66.7 (100.0)	45.1 (40.4)<br>33.3 (75.0)	86.7 (29.4)<br>100.0 (0.0)	F=20.1 (3, 104.0)	**<.001**	*η2*=.18
**Energy/Fatigue (FS n=46, FMS n=45, HC n=49)**	29.6 (19.1)<br>30.0 (28.8)	25.2 (15.7)<br>25.0 (20.0)	34.4 (19.9)<br>35.0 (16.2)	60.7 (14.5)<br>65.0 (15.0)	F=40.0 (3, 184)	**<.001**	*η2*=.39
**Emotional Wellbeing***	52.9 (19.1)<br>52.0 (28.0)	58.2 (22.9)<br>64.0 (36.0)	51.8 (20.7)<br>52.0 (33.0)	78.7 (11.6)<br>80.0 (15.0)	F=36.9 (3, 103.0)	**<.001**	*η2*=.25
**Social Functioning* (FS n=49, FMS n=49)**	39.3 (25.8)<br>37.5 (50.0)	39.0 (26.3)<br>37.5 (50.0)	62.5 (29.3)<br>75.0 (50.0)	90.8 (14.5)<br>100.0 (12.5)	F=80.2 (3, 101.0)	**<.001**	*η2*=.44
**Pain***	41.5 (23.6)<br>42.5 (34.4)	36.6 (22.6)<br>32.5 (26.9)	64.1 (25.2)<br>67.5 (42.5)	82.5 (17.6)<br>90.0 (12.5)	F=55.1 (3, 106.0)	**<.001**	*η2*=.41
**General Health**	36.9 (20.6)<br>35.0 (30.0)	33.3 (22.2)<br>30.0 (30.0)	51.9 (22.0)<br>47.5 (32.5)	75.1 (16.7)<br>77.5 (20.0)	F=43.0 (3, 194)	**<.001**	*η2*=.40
**Work and Social Adjustment Scale***	25.6 (9.4)<br>28.0 (15.8)	26.6 (8.0)<br>28.0 (9.8)	17.5 (10.5)<br>16.0 (17.0)	2.6 (4.4)<br>0.0 (4.8)	F=167.0 (3, 100.0)	**<.001**	*η2*=.58

Notes. *Welch's ANOVA. "No variability in HC group, Identity Dissociation test run with FS, FMS and CC only.

Table S10. Significant self-report clinical measures post hoc tests

Variable	Group comparison	MD	SE	p
**GAD-7**	CC – HC	6.36	[8.59, 4.12]	<.001
	FMS – HC	6.08	[8.59, 3.57]	<.001
	FS – HC	7.33	[10.0, 4.62]	<.001
**PHQ-9**	CC – HC	8.40	[11.1, 5.69]	<.001
	FMS – HC	8.54	[11.1, 5.97]	<.001
	FS – HC	9.83	[12.5, 7.12]	<.001
**PHQ-15**	CC – FMS	-3.95	[-1.49, -6.41]	<.001
	CC – FS	-4.69	[-1.80, -7.58]	<.001
	CC – HC	5.41	[7.57, 3.24]	<.001
	FMS – HC	9.36	[11.4, 7.30]	<.001
	FS – HC	10.1	[12.7, 7.53]	<.001
**WSAS**	CC – FMS	-9.1	[-4.16, -14.0]	<.001
	CC – FS	-8.14	[-2.87, -13.4]	<.001
	CC – HC	14.9	[19.2, 10.6]	<.001
	FMS – HC	24.0	[27.4, 20.6]	<.001
	FS – HC	23.0	[26.9, 19.1]	<.001
**PDS-5**	CC – FS	-16.7	[-5.73, -27.7]	.001
	CC – HC	7.19	[13.6, .82]	.02
	FMS – FS	-12.3	[-.50, -24.0]	.038
	FMS – HC	11.6	[19.3, 3.95]	.001
	FS – HC	23.9	[33.5, 14.3]	<.001
**SDQ-20**	CC – FMS	-8.02	[-4.77, -11.3]	<.001
	CC – FS	-12.0	[-7.40, -16.6]	<.001
	CC – HC	2.42	[4.41, .44]	.01
	FMS – HC	10.4	[13.1, 7.74]	<.001
	FS – HC	14.4	[18.7, 10.2]	<.001
**TEC total**	FMS vs HC	2.36	[4.25, .47]	.008
	FS vs HC	3.68	[6.25, 1.11]	.002
**TEC impact**	FMS vs HC	11	[18.8, 3.21]	.002
	FS vs HC	17.9	[29.3, 6.43]	.001
**MDI-D**	CC vs FMS	-11.7	[-3.20, -20.2]	.003
	CC vs FS	-19.6	[-11.3, -27.8]	<.001
	CC vs HC	11.1	[17.1, 5.12]	<.001
	FMS vs HC	22.8	[30.7, 14.9]	<.001
	FS vs HC	30.7	[38.2, 23.1]	<.001
**MDI-DP**	CC vs FMS	-18.9	[-2.11, -35.6]	.021
	CC vs FS	-26.0	[-8.61, -43.5]	.001
	CC vs HC	15.4	[25.1, 5.64]	<.001
	FMS vs HC	34.2	[48.4, 20.0]	<.001
	FS vs HC	41.4	[56.4, 26.4]	<.001

**MDI-DR**	CC vs FS	-23.5	[-10.4, -36.5]	<.001
	CC vs HC	10.7	[18.8, 2.66]	.005
	FMS vs HC	18.5	[26.5, 10.5]	<.001
	FS vs HC	34.2	[44.8, 23.5]	<.001
	FMS vs FS	-15.7	[-2.62, -28.7]	.012
**MDI-EC**	CC vs FS	-13.2	[-1.13, -25.3]	.026
	CC vs HC	9.60	[17.0, 2.16]	.006
	FMS vs HC	13.9	[22.7, 5.17]	.001
	FS vs HC	22.8	[33.1, 12.5]	<.001
**MDI-MD**	CC vs FMS	-15.3	[-1.70, -28.9]	.021
	CC vs FS	-33.3	[-17.6, -49.0]	<.001
	FMS vs FS	-18.0	[-.77, -35.3]	.037
	FMS vs HC	24.4	[36.1, 12.7]	<.001
	FS vs HC	42.4	[56.6, 28.3]	<.001
**AQ**	CC vs HC	9.35	[13.8, 4.94]	<.001
	FMS vs HC	9.12	[13.5, 4.73]	<.001
	FS vs HC	9.96	[14.6, 5.30]	<.001
**AQ Social Skills**	CC vs HC	2.81	[4.08, 1.55]	<.001
	FMS vs HC	2.96	[4.19, 1.73]	<.001
	FS vs HC	2.86	[4.19, 1.53]	<.001
**AQ Attention Switching**	CC vs HC	1.97	[3.28, .65]	.001
	FMS vs HC	2.16	[3.30, 1.02]	<.001
	FS vs HC	2.03	[3.30, .76]	<.001
**AQ Communication**	CC vs HC	2.28	[3.57, .98]	.001
	FMS vs HC	2.28	[3.46, 1.10]	<.001
	FS vs HC	3.14	[4.42, 1.85]	<.001
**TAS-20**	CC vs FMS	-7.66	[-.94, -14.4]	.019
	CC vs HC	10.3	[16.4, 4.12]	<.001
	FMS vs HC	17.9	[24.4, 11.5]	<.001
	FS vs HC	16.5	[22.8, 10.2]	<.001
**TAS-20 Difficulty Identifying**	CC vs FMS	-3.39	[-.13, -6.65]	.038
	CC vs HC	5.76	[8.62, 2.89]	<.001
	FMS vs HC	9.15	[12.2, 6.09]	<.001
	FS vs HC	8.43	[11.4, 5.47]	<.001
**TAS-20 Difficulty Describing**	CC vs HC	3.02	[5.50, .53]	.011
	FMS vs HC	4.15	[6.71, 1.58]	<.001
	FS vs HC	4.67	[7.16, 2.19]	<.001
**TAS-20 External Orient**	CC vs FMS	-3.04	[-.58, -5.50]	.009
	FMS vs HC	4.56	[7.08, 2.04]	<.001
	FS vs HC	3.43	[6.15, .71]	.007
**SF-36 Physical Functioning**	CC vs FMS	52.5	[65.7, 39.4]	<.001
	CC vs FS	30.8	[44.7, 17.0]	<.001

	CC vs HC	-10.1	[-1.11, -19.0]	.022
	FMS vs FS	-21.7	[-6.93, -36.5]	.001
	FMS vs HC	-62.6	[-52.2, -73.0]	<.001
	FS vs HC	-40.9	[-29.6, -52.2]	<.001
**SF-36 Role Limitations due to Physical Health**	CC vs FMS	57.9	[75.7, 40.0]	<.001
	CC vs FS	55.5	[74.3, 36.8]	<.001
	CC vs HC	-18.6	[-1.06, -36.2]	.033
	FMS vs HC	-76.5	[-63.4, -89.6]	<.001
	FS vs HC	-74.2	[-59.8, -88.5]	<.001
**SF-36 Role Limitations due to Emotional Problems**	CC vs HC	-41.5	[-22.8, -60.3]	<.001
	FMS vs HC	-36.0	[-15.5, -56.4]	<.001
	FS vs HC	-48.7	[-28.8, -68.5]	<.001
**SF-36 Energy/Fatigue**	CC vs HC	-26.3	[-17.0, -35.6]	<.001
	FMS vs HC	-35.5	[-27.3, -43.7]	<.001
	FS vs HC	-31.1	[-22.0, -40.3]	<.001
**SF-36 Emotional Wellbeing**	CC vs HC	-27.0	[-18.0, -35.9]	<.001
	FMS vs HC	-20.5	[-10.9, -30.0]	<.001
	FS vs HC	-25.8	[-17.6, -34.1]	<.001
**SF-36 Social Functioning**	CC vs FMS	23.5	[38.3, 8.66]	<.001
	CC vs FS	23.2	[37.9, 8.55]	<.001
	CC vs HC	-28.3	[-15.9, -40.7]	<.001
	FMS vs HC	-51.8	[-40.5, -63.0]	<.001
	FS vs HC	-51.5	[-40.4, -62.6]	<.001
**SF-36 Pain**	CC vs FMS	27.5	[40.2, 14.9]	<.001
	CC vs FS	22.6	[35.4, 9.68]	<.001
	CC vs HC	-18.4	[-6.89, -30.0]	<.001
	FMS vs HC	-46.0	[-35.5, -56.6]	<.001
	FS vs HC	-41	[-30.1, -51.9]	<.001
**SF-36 General Health**	CC vs FMS	18.6	[30.2, 6.91]	<.001
	CC vs FS	15.0	[26.2, 3.70]	.004
	CC vs HC	-23.2	[-12.9, -33.6]	<.001
	FMS vs HC	-41.8	[-31.5, -52.1]	<.001
	FS vs HC	-38.2	[-28.4, -48.0]	<.001

Notes. All Games-Howell. AQ = Autism Spectrum Quotient; GAD7 = Generalized Anxiety Disorder-7; MDI-D = Multiscale Dissociation Inventory Disengagement; MDI-DP = Multiscale Dissociation Inventory Depersonalisation; MDI-DR = Multiscale Dissociation Inventory Derealisation; MDI-EC = Multiscale Dissociation Inventory Emotional Constriction; MDI-MD = Multiscale Dissociation Inventory Memory Disturbance; MDI-ID = Multiscale Dissociation Inventory Identity Dissociation; PHQ-9 = Patient Health Questionnaire-9; PHQ-15 = Patient Health questionnaire-15; SDQ-20 = Somatoform Dissociation Questionnaire-20; SF36PF = 36-Item Short Form Health Survey; TAS-20 = Toronto Alexithymia Scale-20.

Table S11. Proportion of participants scoring above clinical cut-offs

	FS	FMS	CC	HC			
	n (%)	*n (%)*	*n (%)*	*n (%)*	*χ2*	*p*	*v*
**PHQ-9 (FS n=49)**							
1-4 minimal	8 (16)	9 (18)	9 (19)	40 (80)	65.64	**<.001**	.58
5-9 mild	8 (16)	17 (34)	14 (29)	9 (18)	6.14	.11	.18
10-14 moderate	17 (35)	7 (14)	10 (21)	1 (2)	NA	**.0002^**	.30
15-19 moderately severe	7 (14)	13 (26)	11 (23)	0 (0)	NA	**.0002^**	.28
20-27 severe	9 (18)	4 (8)	4 (8)	0 (0)	NA	**.008^**	.23
**GAD-7 (FS n=47)**							
0-4 minimal	11 (23)	17 (34)	8 (17)	39 (78)	49.06	**<.001**	.50
5-9 mild	18 (38)	13 (26)	19 (40)	8 (16)	8.07	**.045**	.20
10-14 moderate	4 (9)	8 (16)	14 (29)	3 (6)	NA	**.007^**	.26
15-21 severe	14 (30)	12 (24)	7 (15)	0 (0)	NA	**<.001^**	.29
**PDS-5 (28-80 probable PTSD) (FS n=48)**	25 (52)	12 (24)	10 (21)	0 (0)	NA	**<.001^**	.42
**AQ score ≥32 (FS n=49, HC n=49)**	12 (24)	9 (18)	10 (21)	1 (2)	NA	**.004^**	.23
**TAS-20 score ≥61 (FS n=49, HC n=49)**	18 (37)	23 (46)	11 (23)	3 (6)	NA	**<.001^**	.34

Notes. GAD-7 = Generalized Anxiety Disorder-7; PHQ-9 = Patient Health Questionnaire-9; PDS-5 = Posttraumatic Stress Diagnostic Scale – 5; AQ = Autism Spectrum Quotient; TAS-20 = Toronto Alexithymia Scale-20. ^Fisher's Exact Test

Table S12. Significant clinical cut-offs post hoc tests.

Variable	Group comparison	p
**PHQ-9 minimal**	CC vs HC	<.001
	FMS vs HC	<.001
	FS vs HC	<.001
**PHQ-9 moderate**	CC vs HC	.017
	FS vs HC	<.001
**PHQ-9 moderately severe**	CC vs HC	.001
	FMS vs HC	.001
**PHQ9 severe**	FS vs HC	.016
**GAD-7 minimal**	CC vs HC	<.001
	FMS vs HC	<.001
	FS vs HC	<.001
**GAD-7 moderate**	CC vs FS	.044
	CC vs HC	.018
**GAD-7 severe**	CC vs HC	.021
	FMS vs HC	.001
	FS and HC	<.001
**PDS-5**	CC vs FS	.01
	CC vs HC	.002
	FS vs FMS	.025
	FMS vs HC	.001
	FS vs HC	<.001
**AQ**	CC vs HC	.017
	FS vs HC	.012
**TAS-20**	FMS vs HC	<.001
	FS vs HC	.002

Notes. All with Holm correction. GAD-7 = Generalized Anxiety Disorder-7; PHQ-9 = Patient Health Questionnaire-9; PDS-5 = Posttraumatic Stress Diagnostic Scale – 5; AQ = Autism Spectrum Quotient; TAS-20 = Toronto Alexithymia Scale-20. ^Fisher's Exact Test

Table S13. Significant precipitating factors, symptom triggers, and illness causes post hoc tests

Variable	Group comparison	p
**Precipitating factors**		
Physical illness/injury	CC - FMS	<.001
	CC - FS	<.001
	FS - FMS	.044
Traumatic/adverse events	CC - FS	.031
Mental health	CC - FS	.022
**Symptom triggers**		
Physical activity	CC - FMS	<.001
	CC - FS	.028
	FMS - FS	.011
Emotional	CC - FMS	.011
Other physical	CC - FMS	.002
	CC - FS	.001
Sensory	CC - FMS	<.001
	CC - FS	<.001
	FS - FMS	.042
Social	CC - FMS	<.001
	CC - FS	.001
	FMS - FS	.01
Other psychological	CC - FMS	.002
**BIPQ Perceived Causes of Illness**		
Physical	CC - FMS	<.001
	CC - FS	<.001
Mental health	CC - FS	.011
Genetic/familial or psychological vulnerability	CC - FMS	<.001
	CC - FS	<.001
Other physical	FMS - FS	.024
Childhood factors or neurodivergence	CC - FMS	<.001
	CC - FS	.001

Notes. All with Holm correction; BIPQ = Brief Illness Perception Questionnaire.

Table S14. Illness beliefs and cognitive-behavioural responses

	M (*SD*)<br>Mdn (IQR)	M (*SD*)<br>Mdn<br>(IQR)	M (*SD*)<br>Mdn (IQR)	*F* (*df*)	*p*	*η2*
**BIPQ-9**						
**Consequences (FS n=49, CC n=48)**	7.9 (2.07)<br>8.0 (3.0)	8.0 (2.1)<br>8.0 (3.8)	5.60 (2.6)<br>6 (5.0)	16.9 (2, 144)	**<.001**	.19
**Timeline (FS n=46, CC n=48, FMS n=48)**	8.4 (2.1)<br>10 (3.0)	8.5 (2.0)<br>9.5 (3.0)	8.0 (2.9)<br>9 (3.0)	.5 (2, 139)	.60	.01
**Personal Control (FS n=49, CC n=48)**	6.9 (2.7)<br>7.0 (5.0)	6.4 (2.6)<br>7.0 (3.8)	5.4 (2.1)<br>5.0 (3.0)	4.8 (2, 144)	.01	.06
**Treatment Control (FS n=42, CC n=46, FMS n=45)**	5.8 (2.5)<br>6.0 (3.8)	5.0 (3.1)<br>5.0 (4.0)	3.6 (2.6)<br>3.0 (3.0)	7.3 (2, 130)	**.001**	.10
**Identity (FS n=49, CC n=48)**	7.5 (2.0)<br>7.0 (3.0)	7.3 (1.9)<br>7.5 (2.0)	5.5 (2.5)<br>6.0 (4.3)	12.8 (2, 144)	**<.001**	.15
**Concern (FS n=49, CC n=58)**	7.1 (2.2)<br>7.0 (4.0)	6.5 (2.7)<br>7.0 (3.0)	5.0 (2.6)<br>5.0 (4.0)	9.2 (2, 144)	**<.001**	.11
**Coherence (FS n=49, CC n=48)**	4.4 (2.7)<br>5.0 (5.0)	4.3 (3.0)<br>4.0 (4.8)	3.2 (2.3)<br>3.0 (3.0)	2.7 (2, 144)	.07	.04
**Emotional Representation (FS n=49, CC n=48)**	7.5 (2.2)<br>7.0 (4.0)	6.4 (2.5)<br>7.0 (3.8)	6.3 (2.7)<br>7.0 (3.0)	3.3 (2, 144)	.04	.04
**Illness Threat (FS n=46, CC n=48, FMS n=48)**	55.3 (10.0)<br>55.0 (12.5)	52.9<br>(11.3)<br>53.0<br>(16.2)	42.7 (11.9)<br>42.5 (18.2)	17.3 (2, 139)	**<.001**	.20
**CBRQ**						
**Fear Avoidance (FS n=49, CC n=48)**	11.4 (3.9)<br>11.0 (6.0)	13.3 (4.4)<br>13.0 (4.8)	6.9 (4.4)<br>6.5 (6.0)	29.0 (2, 144)	**<.001**	.29
**Catastrophizing (FS n=48, CC n=48)**	8.8 (3.4)<br>8.5 (4.5)	8.9 (3.3)<br>9.0 (4.0)	6.1 (3.5)<br>6.0 (4.0)	11.2 (2, 143)	**<.001**	.14
**Damaging Beliefs (FS n=49, CC n=48)**	11.0 (2.4)<br>11.0 (2.0)	11.2 (2.1)<br>11.0 (2.0)	10.2 (2.0)<br>10.0 (2.0)	2.7 (2, 144)	.074	.04
**Embarrassment Avoidance (FS n=49, CC n=48)**	15.7 (5.4)<br>16.0 (6.0)	14.0 (5.7)<br>14.5 (5.5)	12.5 (5.4)<br>13.0 (7.0)	4.3 (2, 144)	.016	.06
**Symptom Focus (FS n=49, CC n=48)**	14.5 (5.0)<br>15.0 (7.0)	13.8 (5.2)<br>14.0 (7.8)	12.9 (4.8)<br>14.0 (6.0)	1.3 (2, 144)	.28	.02
**All or Nothing (FS n=49, CC n=48)**	13.1 (3.0)<br>13.0 (4.0)	12.6 (4.5)<br>14.0 (4.0)	11.5 (3.8)<br>11.0 (5.3)	2.4 (2, 144)	.09	.03
**Avoidance/Resting Behaviour (FS n=49, CC n=48)**	18.8 (4.3)<br>19.0 (6.0)	17.0 (6.0)<br>17.0 (6.0)	16.5 (5.8)<br>18.0 (7.3)	2.4 (2, 144)	.09	.03

Notes. BIPQ = Brief Illness Perception Questionnaire; CBRQ = Cognitive Behavioural Response Questionnaire; ^Fisher's Exact Test; *Welch's ANOVA.

Table S15. Illness beliefs post hoc tests

Variable	Group comparison	MD	SE	p
**Consequences**	CC vs FMS	-2.36	[-1.22, -3.49]	<.001
	CC vs FS	-2.25	[-1.12, -3.39]	<.001
**Treatment control**	CC vs FS	-2.18	[-.90, -3.46]	<.001
**Identity**	CC vs FMS	-1.80	[-.72, -2.88]	<.001
	CC vs FS	-2.03	[-.93, -3.14]	<.001
**Concern**	CC vs FMS	-1.48	[-.22, -2.74]	.017
	CC vs FS	-2.12	[-.97, -3.28]	<.001
**Illness threat**	CC vs FMS	-10.2	[-4.58, -15.8]	<.001
	CC vs FS	-12.6	[-7.24, -18.0]	<.001
**Fear Avoidance**	CC vs FMS	-6.40	[-4.28, -8.53]	<.001
	CC vs FS	-4.49	[-2.47, -6.51]	<.001
**Catastrophizing**	CC vs FMS	-2.82	[-1.20, -4.43]	<.001
	CC vs FS	-2.77	[-1.12, -4.42]	<.001

Notes. All Games-Howell.

Table S16. Correlations among symptoms, clinical features, and relevant aetiological factors in FS, FMS and CCs.

Variable	FS		FMS		CC	
	**r/rho**	***p***	**r/rho**	***p***	**r/rho**	***p***
**FNSQ Symptom Count**						
BIPQ consequences	.28 s	.05	.56 s	**<.001**	NA	NA
BIPQ concern	.40 s	.005	.27 s	.055	NA	NA
BIPQ treatment control	-.06 p	.70	-.04 s	.77	NA	NA
BIPQ identity	.30 s	.038	.42 s	**.002**	NA	NA
BIPQ illness threat	.16 p	.28	.31 p	.034	NA	NA
CBRQ Fear Avoidance	.34 p	.018	-.03 p	.85	NA	NA
MDI Disengagement	.25 p	.08	.23 p	.10	NA	NA
MDI Depersonalization	.32 s	.022	.21 s	.14	NA	NA
MDI Derealization	.24 s	.10	.23 s	.11	NA	NA
MDI Emotional Constriction	.26 s	.07	-.17 s	.24	NA	NA
MDI Memory Disturbance	.39 s	.005	.18 s	.21	NA	NA
TAS-20	-.002 p	.99	-.02 p	.87	NA	NA
AQ	-.12 p	.38	.12 p	.40	NA	NA
**FNSQ Symptom Severity**						
BIPQ consequences	.24 s	.09	.49 s	**<.001**	NA	NA
BIPQ concern	.38 s	.007	-.03 s	.85	NA	NA
BIPQ treatment control	-.006 s	.97	-.02 s	.92	NA	NA
BIPQ identity	.29 s	.041	.43 s	**.002**	NA	NA
BIPQ illness threat	.17 s	.25	.19 s	.20	NA	NA
CBRQ Fear Avoidance	-.06 s	.68	.04 s	.80	NA	NA
WSAS	.29 s	.042	.40 s	.004	NA	NA
MDI Disengagement	.04 s	.79	.11 s	.47	NA	NA
MDI Depersonalization	.12 s	.39	.24 s	.09	NA	NA
MDI Derealization	.13 s	.37	.15 s	.30	NA	NA
MDI Emotional Constriction	.32 s	.026	.11 s	.44	NA	NA
MDI Memory Disturbance	.16 s	.27	.15 s	.30	NA	NA
TAS-20	-.06 s	.68	-.10 s	.48	NA	NA
AQ	.03 s	.85	.02 s	.89	NA	NA
**FNSQ Symptom Impact**						
BIPQ consequences	.34 s	.019	.45 s	**.001**	NA	NA
BIPQ concern	.43 s	**.002**	-.001 s	.99	NA	NA
BIPQ treatment control	-.01 s	.97	-.04 s	.78	NA	NA
BIPQ identity	.40 s	.004	.34 s	.014	NA	NA
BIPQ illness threat	.29 s	.05	.10 s	.48	NA	NA
CBRQ Fear Avoidance	-.07 s	.61	-.03 s	.84	NA	NA
MDI Disengagement	.01 s	.96	.13 s	.38	NA	NA
MDI Depersonalization	.13 s	.35	.14 s	.33	NA	NA
MDI Derealization	.10 s	.49	.11 s	.47	NA	NA
MDI Emotional Constriction	.22 s	.12	.11 s	.43	NA	NA

MDI Memory Disturbance	.12 s	.39	.18 s	.22	NA	NA
TAS-20	-.16 s	.26	-.16 s	.26	NA	NA
AQ	-.12 s	.41	.14 s	.32	NA	NA
**WSAS**						
PHQ-9	.41 s	**.003**	.41 s	**.003**	.72 s	**<.001**
CBRQ Fear Avoidance	.32 s	**.025**	.21 p	.14	.05 s	.74
**CBRQ Fear Avoidance**						
SF-36 Physical Functioning	-.35 p	.015	-.26 s	.065	-.33 s	.022
SF-36 RL Phys	-.41 s	**.004**	-.08 s	.56	-.35 s	.015
SF-36 RL Emotional	-.07 s	.62	-.48 s	**<.001**	-.25 s	.083
SF-36 Energy	-.30 p	.049	-.44 p	**.003**	-.19 p	.19
SF-36 Emotional Wellbeing	.01 p	.93	-.27 p	.063	-.12 p	.41
SF-36 Social Functioning	-.24 s	.10	-.20 s	.18	-.15 s	.32
SF-36 Pain	-.35 p	.014	-.24 s	.10	-.30 p	.037
SF-36 General Health	-.23 p	.10	-.36 s	.011	-.27	.063
**TEC total**						
SF-36 Physical Functioning	-.06 s	.66	-.31 s	.027	-.40 s	**.005**
SF-36 RL Phys	-.26 s	.070	-.43 s	**.002**	-.33 s	.024
SF-36 RL Emotional	-.52 s	**<.001**	-.14 s	.35	-.27 s	.059
SF-36 Energy	-.01 s	.91	-.17 p	.27	-.22 s	.13
SF-36 Emotional Wellbeing	-.31 s	.026	-.20 p	.15	-.23 s	.12
SF-36 Social Functioning	-.23 s	.11	-.20 s	.17	-.34 s	.017
SF-36 Pain	-.33 s	.019	-.26 s	.070	-.32 s	.028
SF-36 General Health	-.23 s	.11	-.21 s	.15	-.40 s	**.005**

Notes. All Games-Howell. AQ = Autism Spectrum Quotient; BIPQ = Brief Illness Perception Questionnaire; CBRQ = Cognitive Behavioural Response Questionnaire; FNSQ = Functional Neurological Symptoms Questionnaire; MDI= Multiscale Dissociation Inventory; PHQ-9 = Patient Health Questionnaire-9; SDQ-20 = Somatoform Dissociation Questionnaire-20; SF36PF = 36-Item Short Form Health Survey; TAS-20 = Toronto Alexithymia Scale-20; TEC = Traumatic Experiences Scale; WSAS = Work and Social Functioning Scale

Table S17. Self-report questionnaire analyses with outliers winsorized.

	FS	FMS	CC	HC		
Variable	M (*SD*)	M (*SD*)	M (*SD*)	M (*SD*)	*F* (*df*)	*p*
**GAD7 *Welch's ANOVA (FS n=47)**	9.87 (6.41)	8.62 (6.03)	8.90 (5.09)	2.46 (2.79)	37.7 (3, 98.3)	**<.001**
**PHQ9 *Welch's ANOVA (FS n=49)**	12.3 (6.78)	11.0 (6.45)	10.9 (6.68)	2.44 (2.26)	65.9 (3, 93.1)	**<.001**
**PHQ15 *Welch's ANOVA**	14.9 (6.11)	14.2 (4.53)	10.2 (4.62)	4.82 (3.22)	65.4 (3, 105.0)	**<.001**
**WSAS *Welch's ANOVA**	25.6 (9.42)	26.6 (7.97)	17.5 (10.5)	2.44 (3.78)	190.0 (3, 97.4)	**<.001**
**PDS-5 *Welch's ANOVA (FS n=48, FMS n=49)**	27.4 (24.5)	15 (19.0)	10.7 (15.7)	3.26 (5.23)	21.3 (3, 87.7)	**<.001**
**SDQ-20 *Welch's ANOVA (FS n=49)**	34.8 (10.4)	30.9 (6.56)	23.0 (5.05)	20.6 (1.11)	69.7 (3, 84.2)	**<.001**
**PDS-5 Intrusion *Welch's ANOVA (FS n=46, CC n=48)**	6.59 (6.48)<br>6 (11.8)	3.64 (4.75)<br>2 (5)	2.27 (3.29)<br>0 (4.25)	.62 (1.28)<br>0 (.75)	19.8 (3, 88.4)	**<.001**
**PDS-5 Avoidance *Welch's ANOVA (FS n=47, FMS n=48, CC n=48)**	3.30 (2.92)<br>3 (5.5)	1.77 (2.36)<br>1 (3)	1.04 (1.68)<br>0 (2)	.48 (.91)<br>0 (.75)	16.2 (3, 94.1)	**<.001**
**PDS-5 Changes in cognition and mood *Welch's ANOVA (FS n=47, FMS n=49, CC n=48)**	10.1 (9.36)<br>9 (19)	5.22 (7.16)<br>2 (10)	4.12 (6.24)<br>0 (7.5)	.9 (1.73)<br>0 (.75)	22.0 (3, 85.5)	**<.001**
**PDS-5 Arousal and hyperreactivity *Welch's ANOVA (FS n=47, CC n=48)**	8.15 (7.18)<br>9 (14)	4.86 (5.91)<br>1.5 (7)	3.06 (4.78)<br>0 (6)	.96 (1.73)<br>0 (2)	21.4 (3, 89.6)	**<.001**
**TEC total *Welch's ANOVA (CC n=48)**	7.46 (5.81)<br>6 (9.75)	6.16 (3.56)<br>6 (5.75)	5.15 (3.72)<br>5 (4.25)	3.66 (3.23)<br>3 (4.75)	7.42 (3, 106.0)	**<.001**
**TEC impact *Welch's ANOVA (CC n=48)**	29.7 (25.3)<br>20.5 (41)	23.1 (14.4)<br>21 (22.8)	18.2 (13.3)<br>16 (22)	11.8 (12.4)<br>8 (14)	9.7 (3, 106.0)	**<.001**
**MDID** *Welch's ANOVA	84.6 (18.0)<br>84 (27.8)	76.7 (18.9)<br>76 (20)	64.9 (12.5)<br>64 (21)	53.9 (9.50)<br>52 (12)	47.5 (3, 104.0)	**<.001**
**MDIDP** *Welch's ANOVA	89.5 (39.6)<br>82 (53)	82.3 (37.4)<br>74 (53)	61.2 (17.8)<br>51.5 (27)	47.5 (2.16)<br>47 (0)	41.1 (3, 81.6)	**<.001**
**MDIDR** *Welch's ANOVA **(FS n=49, CC n=48)**	81.2 (27.9)<br>74 (33)	65.3 (20.3)<br>60 (31.8)	56.7 (16.6)<br>52 (17)	47.0 (2.22)<br>46 (0)	42.0 (3, 81.8)	**<.001**
**MDIEC** *Welch's ANOVA	71.6 (26.7)<br>64 (35)	62.7 (22.2)<br>50 (30)	57.3 (14.9)<br>50 (18)	48.3 (4.93)<br>46 (0)	21.6 (3, 89.0)	**<.001**
**MDIMD** *Welch's ANOVA	93.3 (36.2)<br>83 (56)	75.2 (29.1)<br>64 (38)	59.4 (19.4)<br>53 (21.5)	50.3 (8.27)<br>45 (7)	32.5 (3, 92.8)	**<.001**
**MDIID** *Welch's ANOVA	62.5 (29.6)<br>47 (12)	56.5 (22.7)<br>47 (0)	51.3 (10.8)<br>47 (0)	*No variability, Welch's ANOVA will not run – MDIID test run with FS, FMS and CC only<br>47 (0)<br>47 (0)	3.67 (2, 83.2)	.03

**AQ total *Welch's ANOVA (FS n=49, CC n=48, HC n=49)**	24.8 (10.3)<br>23 (13)	23.9 (9.61)<br>23 (12)	24.2 (9.46)<br>24 (13)	14.7 (6.47)<br>14 (10)	20.3 (3, 105.0)	**<.001**
**AQ Social Skills *Welch's ANOVA (FS n=49, FMS n=49, CC n=48, HC n=49)**	4.84 (2.95)<br>5 (4)	4.94 (2.64)<br>5 (4)	4.79 (2.72)<br>5 (4.25)	1.96 (1.91)<br>1 (3)	21.8 (3, 104.0)	**<.001**
**AQ Attention Switching (FS n=48, CC n=48)**	6.27 (2.66)<br>6.5 (3)	6.4 (2.26)<br>6 (3.75)	6.21 (2.80)<br>7 (4)	4.24 (2.12)<br>4 (2)	8.66 (3, 192)	**<.001**
**AQ Attention to Detail (FS n=49, CC n=48, HC n=49)**	5.31 (2.42)<br>5 (3)	5.16 (2.34)<br>5 (3)	5.96 (2.32)<br>5.5 (4)	4.63 (2.19)<br>5 (3)	2.69 (3, 192)	.048
**AQ Communication *Welch's ANOVA (FS n=49, CC n=48)**	4.78 (2.89)<br>5 (4)	3.92 (2.59)<br>3 (4)	3.92 (2.89)<br>3 (4)	1.6 (1.76)<br>1 (2)	20.2 (3, 104.0)	**<.001**
**AQ Imagination *Welch's ANOVA (FS n=49, CC n=48, HC n=49)**	3.94 (3.06)<br>3 (3)	3.56 (2.56)<br>3 (3)	3.29 (2.10)<br>3 (2)	2.33 (1.85)<br>2 (3)	4.67 (3, 105.0)	**.004**
**TAS-20 Total (FS n=49, CC n=48, HC n=49)**	55.6 (12.8)<br>57 (18)	57.1 (13.4)<br>58 (20.8)	49.4 (12.0)<br>47.5 (19.2)	39 (10.6)<br>37 (11)	22.10 (3, 192)	**<.001**
**TAS-20 Difficulty Identifying Emotions (FS n=49, CC n=48, HC n=49)**	20.8 (6.13)<br>22 (6)	21.6 (6.57)<br>21 (11.8)	18.2 (5.76)<br>18.5 (10)	12.3 (4.64)<br>11 (6)	25.76 (3, 192)	**<.001**
**TAS-20 Difficulty Describing Emotions (FS n=49, CC n=48, HC n=49)**	15.4 (4.80)<br>17 (6)	14.9 (5.16)<br>16 (8.75)	13.8 (4.76)<br>13.5 (7)	10.7 (4.50)<br>10 (7)	9.43 (3, 192)	**<.001**
**TAS-20 Externally Oriented**	19.4 (5.20)<br>20 (7)	20.5 (4.47)<br>21 (6.75)	17.2 (4.08)<br>17 (6)	15.9 (4.90)<br>16 (7)	9.86 (3, 192)	**<.001**
**SF36 Physical Functioning *Welch's ANOVA (FS n=49, CC n=48)**	55 (29.1)<br>55 (50)	33.3 (27.1)<br>25 (38.8)	85.8 (22.6)<br>97.5 (16.2)	96.2 (5.40)<br>100 (5)	114.0 (3, 86.0)	**<.001**
**SF36 Role Limitations due to Physical Health *Welch's ANOVA (FS n=49, CC n=48)**	14.3 (24.5)<br>0 (25)	13.5 (24.3)<br>0 (25)	71.9 (39.8)<br>100 (50)	92.5 (17.7)<br>100 (0)	169.0 (3, 104.0)	**<.001**
**SF36 Role Limitations due to Emotional Health *Welch's ANOVA (FMS n=48, CC n=48)**	38.0 (44.7)<br>0 (100)	50.7 (45.6)<br>66.7 (100)	45.1 (40.4)<br>33.3 (75.0)	88.7 (23.9)<br>100 (0)	27.0 (3, 101.0)	**<.001**
**SF36 Energy/Fatigue (FS n=46, FMS n=45, CC n=48, HC n=49)**	29.6 (19.1)<br>30 (28.8)	25.1 (15.4)<br>25 (20)	34.4 (19.9)<br>35 (16.2)	60.7 (14.5)<br>65 (15)	40.36 (3, 184)	**<.001**
**SF36 Emotional Wellbeing *Welch's ANOVA (CC n=48)**	52.9 (19.1)<br>52 (28)	58.2 (22.9)<br>64 (36)	51.8 (20.7)<br>52 (33)	78.7 (11.6)<br>80 (15)	36.9 (3, 103.0)	**<.001**
**SF36 Social Functioning *Welch's ANOVA (FS n=49, FMS n=49, CC n=48)**	39.3 (25.8)<br>37.5 (50)	39.0 (26.3)<br>37.5 (50)	62.5 (29.3)<br>75 (50)	91.3 (12.9)<br>100 (12.5)	89.4 (3, 98.9)	**<.001**
**SF36 Pain *Welch's ANOVA (CC n=48)**	41.5 (23.6)<br>42.5 (34.4)	36.6 (22.6)<br>32.5 (26.9)	64.1 (25.2)<br>67.5 (42.5)	84.0 (13.3)<br>90 (12.5)	75.9 (3, 103.0)	**<.001**
**SF36 General Health (CC n=48)**	36.9 (20.6)<br>35 (30)	33.2 (21.9)<br>30 (30)	51.9 (22.0)<br>47.5 (32.5)	75.6 (15.4)<br>77.5 (20)	45.62 (3, 194)	**<.001**
**BIPQ Consequences (FS n=49, CC n=48)**	7.90 (1.95)<br>8 (3)	8 (1.97)<br>8 (3.75)	5.60 (2.59)<br>6 (5)	NA	18.64 (2, 144)	**<.001**
**BIPQ Timeline (FS n=46, CC n=48, FMS n=48)**	8.37 (2.13)<br>10 (3)	8.46 (2)<br>9.5 (3)	8.04 (2.66)<br>9 (3)	NA	.44 (2, 139	.64

**BIPQ Identity (FS n=49, CC n=48) *Welch's ANOVA**	7.59 (1.78)<br>7 (3)	7.28 (1.93)<br>7.5 (2)	5.48 (2.51)<br>6 (4.25)	NA	12.0 (2, 93.9)	**<.001**
**BIPQ Concern (FS n=49, CC n=58)**	7.18 (2.05)<br>7 (4)	6.48 (2.69)<br>7 (3)	5 (2.55)<br>5 (4)	NA	10.05 (2, 144)	**<.001**
**BIPQ Coherence (FS n=49, CC n=48)**	4.37 (2.68)<br>5 (5)	4.26 (2.98)<br>4 (4.75)	3.15 (2.11)<br>3 (3)	NA	3.23 (2, 144)	.043
**BIPQ Symptom Focus (FS n=49, CC n=48)**	14.5 (4.95)<br>15 (7)	13.8 (5.16)<br>14 (7.75)	13.1 (4.25)<br>14 (6)	NA	1.04 (2, 144)	.36
**BIPQ All or Nothing *Welch's ANOVA (FS n=49, CC n=48)**	13.2 (2.95)<br>13 (4)	12.6 (4.40)<br>14 (4)	11.5 (3.84)<br>11 (5.25)	NA	2.99 (2, 93.3)	.055

Notes. AQ = Autism Spectrum Quotient; GAD7 = Generalized Anxiety Disorder-7; MDI-D = Multiscale Dissociation Inventory Disengagement; MDI-DP = Multiscale Dissociation Inventory Depersonalisation; MDI-DR = Multiscale Dissociation Inventory Derealisation; MDI-EC = Multiscale Dissociation Inventory Emotional Constriction; MDI-MD = Multiscale Dissociation Inventory Memory Disturbance; MDI-ID = Multiscale Dissociation Inventory Identity Dissociation; PHQ-9 = Patient Health Questionnaire-9; PHQ-15 = Patient Health questionnaire-15; SDQ-20 = Somatoform Dissociation Questionnaire-20; SF36PF = 36-Item Short Form Health Survey; TAS-20 = Toronto Alexithymia Scale-20.

Table S18. ANCOVAs for primary self-report measures across all participant groups, adjusted for education, employment, ethnicity, physical health diagnosis, other current treatment.

**Measure**	**FS EMM (SE)**	**FMS EMM (SE)**	**CC EMM (SE)**	**HC EMM (SE)**	**Test statistics (Group)**
**Generalized Anxiety disorder-7 (FS n=47)**	10.64 (.99)	9.38 (.92)	9.71 (.97)	3.50 (1.09)	F=14.80 (3, 186), p<.001, *η2p*=.24
**Patient Health Questionnaire-(FS n=49)**	12.72 (1.06)	11.48 (.99)	12.20 (1.05)	4.47 (1.18)	F=18.10 (3, 188), p<.001, *η2p* =.33
**Patient Health Questionnaire-15**	15.30 (.83)	14.07 (.78)	11.43 (.82)	6.96 (.92)	F=23.58 (3, 189), p<.001, *η2p* =.45
**Posttraumatic Diagnostic Scale-5 (FS n=48, FMS n=49)**	27.64 (3.25)	14.45 (3.03)	10.92 (3.18)	5.62 (3.57)	F=11.28 (3, 185), p<.001, *η2p* =.21
**Somatoform Dissociation Questionnaire-20 (FS n=49)**	35.40 (1.30)	31.10 (1.21)	24.30 (1.28)	22.60 (1.43)	F=25.64 (3, 188), p<.001, *η2p* =.42
**Traumatic Experiences Checklist total**	7.88 (.77)	6.08 (.72)	5.12 (.77)	4.20 (.86)	F=5.23 (3, 189), p=.002, *η2p* =.10
**Traumatic Experiences Checklist impact**	30.70 (3.32)	22.10 (3.13)	17.80 (3.31)	12.90 (3.71)	F=6.43 (3, 189), p<.001, *η2p* =.12
**Multiscale Dissociation Inventory**					
Disengagement	86.40 (2.75)	77.80 (2.60)	67.80 (2.74)	58.60 (3.08)	F=22.82 (3, 189), p<.001, *η2p* =.38
Depersonalization	91.10 (5.48)	81.70 (5.18)	62.70 (5.47)	48.20 (6.13)	F=14.23 (3, 189), p<.001, *η2p* =.23
Derealization (FS n=49)	83.60 (3.75)	67.20 (3.51)	60.50 (3.71)	50.60 (4.17)	F=17.55 (3, 188), p<.001, *η2p* =.28
Emotional Constriction	73.40 (3.64)	65.80 (3.43)	59.80 (3.63)	49.70 (4.07)	F=9.11 (3, 189), p<.001, *η2p* =.15
Memory Disturbance	94.10 (4.78)	76.90 (4.51)	63.40 (4.76)	55.90 (5.34)	F=15.04 (3, 189), p<.001, *η2p* =.28
Identity Dissociation	68.90 (4.79)	59.80 (4.52)	53.50 (4.78)	48.80 (5.36)	F=4.02 (3, 189), p=.008, *η2p* =.08
**Autism Spectrum Quotient**					
Total (FS n=49, HC n=49)	25.20 (1.69)	24.80 (1.58)	24.00 (1.67)	14.50 (1.88)	F=11.77 (3, 187), p<.001, *η2p* =.17
Social Skills (FS n=49, FMS n=49, HC n=49)	5.24 (.48)	5.44 (.45)	5.01 (.47)	2.22 (.53)	F=12.79 (3, 186), p<.001, *η2p* =.20
Attention Switching (FS n=48)	6.35 (.47)	6.49 (.43)	6.25 (.45)	4.32 (.51)	F=6.47 (3, 187), p<.001, *η2p* =.12
Communication (FS n=49)	5.16 (.48)	4.36 (.44)	4.01 (.47)	1.75 (.52)	F=12.15 (3, 188), p<.001, *η2p* =.18
**Toronto Alexithymia Scale-20**					
Total (FS n=49, HC n=49)	58.20 (2.26)	59.20 (2.12)	50.50 (2.23)	41.00 (2.51)	F=16.24 (3, 187), p<.001, *η2p* =.26
Difficulty Identifying Emotions (FS n=49, HC n=49)	22.20 (1.07)	22.60 (1.00)	19.00 (1.06)	13.70 (1.19)	F=17.43 (3, 187), p<.001, *η2p* =.29
Difficulty Describing Emotions (FS n=49, HC n=49)	16.10 (.89)	15.50 (.84)	14.00 (.88)	11.20 (.99)	F=7.14 (3, 187), p<.001, *η2p* =.13
Externally Oriented Thinking	19.90 (.89)	21.10 (.84)	17.50 (.88)	16.00 (.99)	F=7.65 (3, 187), p<.001, *η2p* =.12
**36-Item Short Form Health Survey**					
Physical Functioning (FS n=49)	59.60 (3.86)	38.90 (3.61)	81.60 (3.82)	87.20 (4.30)	F=38.92 (3, 188), p<.001, *η2p* =.59
Role Limitations due to Physical Health (FS n=49)	16.10 (5.19)	14.90 (4.87)	65.60 (5.16)	77.60 (5.79)	F=44.87 (3, 188), p<.001, *η2p* =.60
Role Limitations due to Emotional Health (FMS n=48)	31.90 (7.32)	47.00 (7.07)	33.30 (7.24)	69.30 (8.16)	F=8.33 (3, 187), p<.001, *η2p*=.19
Energy/Fatigue (FS n=46, FMS n=45, HC n=49)	30.30 (3.23)	26.60 (3.14)	31.10 (3.15)	54.80 (3.58)	F=20.14 (3, 179), p<.001, *η2p*=.41
Emotional Wellbeing	50.30 (3.44)	55.30 (3.25)	47.90 (3.43)	73.90 (3.85)	F=16.43 (3, 189), p<.001, *η2p*=.26

Social Functioning (FS n=49, FMS n=49)	39.60 (4.42)	39.10 (4.19)	57.80 (4.39)	84.00 (4.93)	F=26.66 (3, 187), p<.001, $\eta 2p$=.45
Pain	41.50 (3.97)	37.60 (3.75)	59.50 (3.96)	74.90 (4.45)	F=21.13 (3, 189), p<.001, $\eta 2p$=.43
General Health	37.40 (3.36)	35.30 (3.17)	48.50 (3.35)	66.40 (3.76)	F=20.74 (3, 189), p<.001, $\eta 2p$=.45
**Work and Social Adjustment Scale**	25.30 (1.47)	26.40 (1.39)	19.70 (1.47)	5.80 (1.65)	F=48.84 (3, 189), p<.001, $\eta 2p$=.60

Table S19. ANCOVAs for primary self-report measures across clinical groups (FS, FMS, CC), adjusted for education, employment, ethnicity, physical health diagnosis, other current treatment, symptom duration, current psychotropic medication.

	**FS EMM (SE)**	**FMS EMM (SE)**	**CC EMM (SE)**	**Test statistics**
**BIPQ-9**				
Consequences (FS n=49, CC n=48)	7.77 (.40)	8.17 (.38)	6.49 (.41)	**Group:** F=5.45 (2, 137), p=.005, $\eta 2p$=.23
Treatment Control (FS n=42, CC n=46, FMS n=45)	6.57 (.53)	5.25 (.50)	4.85 (.53)	**Group:** F=4.08 (2, 123), p=.02, $\eta 2p$=.12
Identity (FS n=49, CC n=48)	7.53 (.39)	7.58 (.38)	6.03 (.40)	**Group:** F=5.67 (2, 137), p=.004, $\eta 2p$=.17
Concern (FS n=49, CC n=58)	6.87 (.47)	6.46 (.45)	5.24 (.48)	**Group:** F=3.81 (2, 137), p=.02, $\eta 2p$=.12
Illness Threat (FS n=46, CC n=48, FMS n=48)	56.70 (2.07)	54.20 (1.96)	46.70 (2.06)	**Group:** F=7.61 (2, 132), p=.001, $\eta 2p$=.23
**CBRQ**				
Fear Avoidance (FS n=49, CC n=48)	10.97 (.83)	12.83 (.79)	7.92 (.84)	**Group:** F=10.66 (2, 137), p<.001, $\eta 2p$=.30
Catastrophizing (FS n=48, CC n=48)	8.01 (.64)	8.10 (.61)	6.38 (.65)	**Group:** F=2.63 (2, 136), p=.08, $\eta 2p$=.15
				**Psychotropic Medication:** F=5.83 (2, 136), **p=.02**, $\eta 2p$=.04

Notes. BIPQ-9 = Brief Illness Perception Questionnaire-9; CBRQ = Cognitive Behavioural Response Questionnaire.

Figure S1. Correlation matrix of included self-report measures.

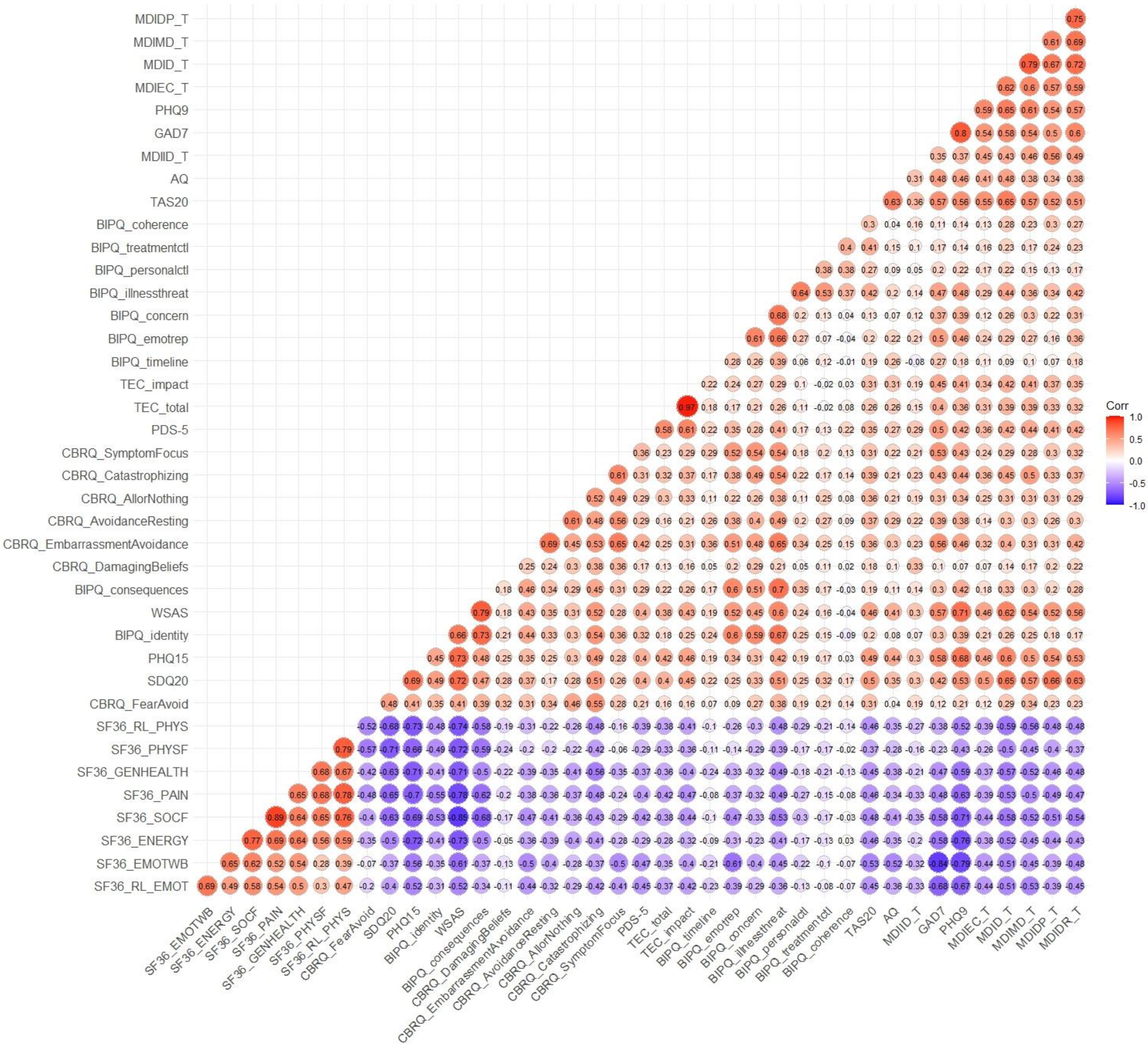

Notes. AQ = Autism Spectrum Quotient; GAD7 = Generalized Anxiety Disorder-7; MDI-D = Multiscale Dissociation Inventory Disengagement; MDI-DP = Multiscale Dissociation Inventory Depersonalisation; MDI-DR = Multiscale Dissociation Inventory Derealisation; MDI-EC = Multiscale Dissociation Inventory Emotional Constriction; MDI-MD = Multiscale Dissociation Inventory Memory Disturbance; MDI-ID = Multiscale Dissociation Inventory Identity Dissociation; PHQ-9 = Patient Health Questionnaire-9; PHQ-15 = Patient Health questionnaire-15; SDQ-20 = Somatoform Dissociation Questionnaire-20; SF36PF = 36-Item Short Form Health Survey; TAS-20 = Toronto Alexithymia Scale-20.
